# Nonfilament-forming RecA dimer catalyzes homologous joint formation

**DOI:** 10.1093/nar/gky877

**Published:** 2018-10-04

**Authors:** Takeshi Shinohara, Naoto Arai, Yukari Iikura, Motochika Kasagi, Tokiha Masuda-Ozawa, Yuuki Yamaguchi, Kayo Suzuki-Nagata, Takehiko Shibata, Tsutomu Mikawa

**Affiliations:** 1 Cellular & Molecular Biology Laboratory, RIKEN, 2-1 Hirosawa, Wako-shi, Saitama 351-0198, Japan; 2 RIKEN Center for Sustainable Resource Science, 2-1 Hirosawa, Wako-shi, Saitama 351-0198, Japan; 3 Department of Supramolecular Biology, Graduate School of Nanobiosciences, Yokohama City University, 1-7-29 Suehiro-cho, Tsurumi-ku, Yokohama, Kanagawa 230-0045, Japan; 4 Department of Applied Biological Science, Nihon University College of Bioresource Sciences, 1866 Kameino, Fujisawa-shi, Kanagawa 252-0880, Japan; 5 RIKEN Quantitative Biology Center, 1-7-22 Suehiro-cho, Tsurumi-ku, Yokohama, Kanagawa 230-0045, Japan; 6 Department of Chemistry, Graduate School of Science, Tokyo Metropolitan University, Minami-Osawa 1-1, Hachioji-shi, Tokyo 192-0397, Japan; 7 RIKEN Center for Biosystems Dynamics Research, 1-7-22 Suehiro-cho, Tsurumi-ku, Yokohama, Kanagawa 230-0045, Japan

## Abstract

Homologous recombination is essential to genome maintenance, and also to genome diversification. In virtually all organisms, homologous recombination depends on the RecA/Rad51-family recombinases, which catalyze ATP-dependent formation of homologous joints—critical intermediates in homologous recombination. RecA/Rad51 binds first to single-stranded (ss) DNA at a damaged site to form a spiral nucleoprotein filament, after which double-stranded (ds) DNA interacts with the filament to search for sequence homology and to form consecutive base pairs with ssDNA (‘pairing’). How sequence homology is recognized and what exact role filament formation plays remain unknown. We addressed the question of whether filament formation is a prerequisite for homologous joint formation. To this end we constructed a nonpolymerizing (np) head-to-tail-fused RecA dimer (npRecA dimer) and an npRecA monomer. The npRecA dimer bound to ssDNA, but did not form continuous filaments upon binding to DNA; it formed beads-on-string structures exclusively. Although its efficiency was lower, the npRecA dimer catalyzed the formation of D-loops (a type of homologous joint), whereas the npRecA monomer was completely defective. Thus, filament formation contributes to efficiency, but is not essential to sequence-homology recognition and pairing, for which a head-to-tail dimer form of RecA protomer is required and sufficient.

## INTRODUCTION

Homologous recombination is conserved in all organisms. It plays an essential role in maintaining genome integrity through the repair of double-strand breaks and in genetic diversification through meiotic recombination, which is required for gametogenesis in sexual reproduction (1–3). In all nuclear genome, homologous recombination depends on the RecA/Rad51-family recombinases. In addition, the *Rad51* gene is essential to vertebrate-cell proliferation (4). The RecA/Rad51-family recombinases catalyze ATP-dependent homologous joint formation. In this reaction, a single-stranded (ss) tail generated by resection at a double-strand break forms base pairs with the complementary strand of the homologous sequence within the intact double-stranded (ds) DNA. The paired tail works as a primer in repair DNA synthesis to copy the complementary strand, thus recovering the sequence lost by the breakage (see (5) for references). How sequence homology between ssDNA and dsDNA is recognized and paired is a critical question. Although many details of RecA/Rad51-catalyzed homologous joint formation have long been studied, this question remains unanswered.

*Escherichia coli* RecA (6,7) is the prototype of the RecA/Rad51-family recombinases, which include eubacterial RecA, Rad51 (8,9) and meiosis-specific Dmc1 (10) for eukaryotes. The RecA/Rad51-family recombinases exhibit a DNA-dependent ATPase activity (11). All RecA/Rad51-family recombinases form a right-handed spiral filament, consisting of about 6 protomers and 18 nucleotides or base pairs per helical turn, around ss- or dsDNA. In the presence of ATP or an ATP analogue, RecA/Rad51-family recombinases extend the length of the DNA in the filament by 1.5-fold, compared to B-form dsDNA with the same base-sequence (12–14).

**Figure 1.**
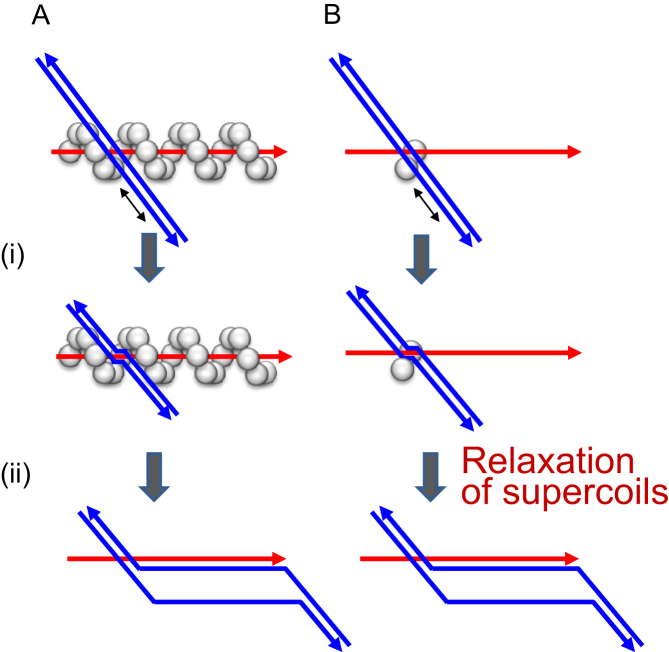
Homologous joint formation by RecA involves two reactions. (**A**) Homologous joint formation by normal RecA. (i) Homologous (DNA) pairing is an ATP-dependent but ATP-hydrolysis-independent reaction. RecA forms spiral filaments around ssDNA, after which free dsDNA interacts with the filaments. Homologous sequences between dsDNA and ssDNA in the filaments are searched for by reiterative association and dissociation and scanning to a limited length, and a homologous-joint nucleus (nascent homologous joint) is formed. The minimum detectable homologous joint is 6–8 bp in length. (ii) Branch migration is an ATP-hydrolysis-dependent reaction. The nascent homologous joints are extended by RecA/Rad51 and can be thousands of base pairs long. (**B**) Assumed homologous joint formation by a pair of adjacent RecA protomers without involvement of the RecA filament. When substrate dsDNA has negative supercoils like natural dsDNA, if a nascent homologous joint is formed, the relaxation of the supercoils promotes the extension of the nascent homologous joints in a protein-independent manner to stabilize the joint. The red lines and the double blue lines represent ssDNA and dsDNA, respectively. RecA is represented by the white balls. The DNA topology is abbreviated in these diagrams.

Homologous joint formation occurs in two phases; homologous (DNA) pairing and subsequent branch migration (Figure 1; (15,16)). In RecA/Rad51-catalyzed homologous joint formation between ssDNA and dsDNA, ATP-bound RecA/Rad51 binds along ssDNA (17) using the primary DNA-binding site that faces the inside of the spiral filament (18–21) to form extended (‘active’) ssDNA filaments (22). This ssDNA binding activates the secondary DNA-binding site of the RecA, to which dsDNA binds independent of sequence homology to ssDNA at the primary DNA-binding site (17). Unlike the primary DNA-binding site, the secondary DNA-binding sites are located within the clefts between adjacent RecA protomers in the filament (23–25). The ATP-bound RecA then promotes homology search between the two DNAs within the ternary complex, consisting of RecA, ssDNA, and dsDNA (Figure 1A (i); (17,26)), resulting in the formation of a homologous-joint nucleus (nascent homologous joint), without ATP hydrolysis (15). This homologous pairing is followed by RecA-promoted branch migration associated with ATP hydrolysis (Figure 1A (ii); (15,16)). The branch migration by RecA has 5′ to 3′ polarity relative to the displaced strand, and is detected *in vitro* as the extension of homologous joints between circular ssDNA and linear dsDNA to >1000 bp (15,16).

The minimum detectable homologous joint formed by RecA/Rad51 is 6–8 bp in length (27–29) without mismatched base pairs (30). The joint is extended in 3 bp increments (29,31), and the minimum stable joint, in the absence of ATP hydrolysis, is 15 bp (29). The 3 bp increment correlates to the triplet structure found in DNA (ss or ds) bound to the extended RecA/Rad51–DNA filaments (18–20). Thus, it is likely that RecA/Rad51 spiral filaments play a role in the extension (and thus the stabilization) of homologous joints.

To resolve the fundamental question of how the homologous sequence is recognized between ssDNA and dsDNA in the RecA/Rad51 spiral filaments, we need to answer the following question: is the initial formation of the 6–8 bp homologous joint and its extension to a stable joint one inseparable process, or can it be separated into two sequential and independent processes? Each RecA/Rad51 protomer accommodates three nucleotide (nt) ssDNA or 3 bp dsDNA. Since the minimum homologous joint is 6–8 bp, as described above, this suggests that two adjacent protomers in the RecA/Rad51-nucloprotein filament are sufficient to form a homologous joint.

In some viruses and mitochondria and in special cases of somatic nuclear recombination, proteins that play an essential role in homologous recombination *in vivo* catalyze ATP-independent homologous joint formation *in vitro* without filament formation (see Discussion). This fact raises further doubt regarding the necessity of filament formation by RecA/Rad51 for sequence-homology recognition and pairing. We thus explored the possibility that homologous joint formation is catalyzed by RecA variants that do not form filaments.

Nascent homologous joints containing only 6–8 bp are unstable and occur transiently. To detect such unstable nascent homologous joints in the absence of RecA-nucleoprotein filament-formation, we relied on the fact that in uncatalyzed homologous pairing detected by D-loop formation of homologous ssDNA and negatively supercoiled dsDNA, once the nucleus of a D-loop, a type of homologous joint, is formed, the D-loop is stabilized by the relaxation of the supercoils (32). Using this D-loop formation with negatively supercoiled dsDNA and homologous ssDNA, we addressed the possibility that homologous joint formation could be catalyzed by nonfilament-forming RecA dimers (Figure 1B).

While a nonpolymerizing (np) RecA monomer was defective in ssDNA binding, a npRecA dimer was active in ssDNA binding, but did not form a nucleoprotein filament on DNA. The npRecA dimer catalyzed D-loop formation. We thus conclude that filament formation is not essential to sequence-homology recognition and pairing, for which two adjacent RecA protomers as a dimer are required and sufficient.

## MATERIALS AND METHODS

### Buffers

The TE0.1 buffer comprised Tris–HCl (10 mM; pH 8.0) and EDTA (0.1 mM). The TE-buffer comprised Tris–HCl (10 mM; pH 8.0) and EDTA (1 mM).

The TEMG buffer consisted of either Tris–HCl (50 mM; pH 7.5), EDTA (1 mM), 2-mercaptoethanol (5 mM) and 5% (w/v) glycerol (TEMG5) for gel filtration, or Tris–HCl (25 mM; pH 7.5), EDTA (1 mM), 2-mercaptoethanol (5 mM), and 10% (w/v) glycerol (TEMG10) for affinity-column purification. The TEM buffer consisted of Tris–HCl (25 mM; pH 7.5), EDTA (1 mM), 2-mercaptoethanol (5 mM). The PEMG buffer consisted of potassium phosphate (50 mM; pH 6.8), 2-mercaptoethanol (5 mM) and 10% (w/v) glycerol. The TMS buffer consisted of Tris–HCl (50 mM; pH 8.0) and 2-mercaptoethanol (5 mM) and 25% (w/v) sucrose.

### Standard reaction buffer

Unless otherwise stated, a standard reaction buffer consisting of Tris–HCl (31 mM; pH 7.5), MgCl_2_ (13 mM), dithiothreitol (DTT; 1.8 mM) and bovine serum albumin (88 μg/ml) was used, as described previously (21,33).

The indicated concentrations of substrates and proteins represent the final concentrations in the reaction mixtures at the initiation of observed reactions, unless otherwise stated.

### DNA for biochemical assays

Concentrations of DNA are expressed in nucleotides.

Note that negatively supercoiled dsDNA prepared by any method, including a denaturation-renaturation process should not be used for the D-loop assay described below, since the unusual structures in the renatured DNA would generate erroneous results.

Negatively supercoiled closed circular dsDNA from pBluescript SK(–) and pKF18 was prepared by a method that included the gentle lysis of *E. coli* cells followed by sucrose-density gradient centrifugation, as described previously (see (21)). *Hin*cII-treated linear dsDNA was treated with TE-buffer-saturated phenol after restriction-endonuclease treatment, according to the manufacturer's recommendations, and then dialyzed against TE0.1.

The OL2 oligo ssDNA was the 90-mer of 5′-AAATCAATCT AAAGTATATA TGAGTAAACT TGGTCTGACAGTTACCAATG CTTAATCAGT GAGGCACCTA TCTCAGCGAT CTGTCTATTT-3′ (21). The Km90 oligo ssDNA was the 90-mer of 5′-ATCTGATCCT TCAACTCAGC AAAAGTTCGA TTTATTCAAC AAAGCCACGT TGTGTCTCAA AATCTCTGAT GTTACATTGC ACAAGATAAA-3′. These oligomers were purchased from Eurofins Genomics K.K. (Tokyo, Japan). The pBluescript SK(-) negatively supercoiled dsDNA contains a region homologous to the OL2 oligo ssDNA, but the pKF18 negatively supercoiled dsDNA does not. The pBluescript SK(–) dsDNA does not contain a region homologous to the Km90 oligo ssDNA, but the pKF18 dsDNA does.

The 5′ termini of these oligo ssDNAs were labeled with ATP [γ-^33^P] using MEGALABEL (TAKARA). The [^33^P] oligo ssDNA was purified with MicroSpin G-25 columns (GE Healthcare), as described previously (21). We estimated the concentrations of the 90-mer oligo ssDNA by assuming that 100% of the oligo ssDNA was recovered.

The FAM-labeled OL2 oligo ssDNA (90-mer) was purchased from Eurofins Genomics K.K. (Tokyo, Japan).

6-Methylisoxanthopterin (6-MI) is a fluorescent guanine analogue that does not affect the Tm of 6-MI-labeled dsDNA (52). 6-MI-labeled 42-mer oligo ssDNA (6-MI oligo ssDNA) is the 42 mer of 5′ CGG TGT GAT TGA TAC XCA CTG CAT ATC GTA ACG GCC TCT CGC 3′, in which X is 6-MI. We purchased this oligomer from TriLink Biotechnologies (San Diego, CA. USA).

### Purification of npRecA dimer

The npRecA dimer concentration, consisting of two RecA subunits, was expressed as the concentration of RecA subunits.

The following procedures were performed on ice or at 4°C. The frozen transformed cells (3 g) were thawed at 4°C and suspended in TMS buffer (30 ml). Lysozyme (0.25–0.6 mg/ml) was added to the cell suspension, and the mixture was incubated for 30 min. 0.5% (w/v) Brij 58 was then added to the mixture, which was incubated for an additional 30 min. TMS buffer (60 ml) was next added to the mixture, followed by the addition of ammonium sulfate to 3–5% saturation over a 10 min period, with stirring. The mixture was stirred for an additional 30 min, then centrifuged at 60 000 × g for 60 min, after which the supernatants were saved. 0.3% (w/v) polyethyleneimine (Polymin P) was added to the supernatants over a 15 min period, with stirring. Stirring continued for another 30 min. The mixture was centrifuged at 13 420 × g for 10 min. The supernatants were saved. Ammonium sulfate was added to the supernatants to 15% saturation, and the mixture was applied to a TOYOPEARL Butyl-650M column (TOSOH). The column was washed with TEMG10-AS15 buffer (TEMG10 supplemented with ammonium sulfate at 15% saturation; 10–15 column volumes), and the npRecA dimers were eluted with a linear gradient of ammonium sulfate from 15% saturation to 0% saturation, in TEMG buffer (10 column volumes). The peak fractions of the npRecA dimer were collected and dialyzed overnight against PEMG-50 mM KCl buffer (PEMG supplemented with KCl [50 mM]). The protein solution was then applied to a P11 column (Whatman), which was washed with a linear gradient of KCl from 0 to 100 mM in PEMG buffer (three column volumes), at which point the KCl concentration was maintained at 100 mM. The npRecA dimer was eluted from the column at this stage. The peak fractions of the npRecA dimers were collected, concentrated and diluted with TEM buffer to decrease the KCl concentration for MonoQ 5/50 GL column chromatography. The protein solution was applied to a MonoQ 5/50 GL column (GE Healthcare), which was eluted with a linear gradient of KCl from 0 to 500 mM in TEM buffer (20 column volumes). Each fraction containing npRecA dimers was tested for nuclease contamination, under conditions for a D-loop assay. The nuclease-free fractions were collected, concentrated, dialyzed against buffer containing Tris–HCl (20 mM; pH 7.5), EDTA (1 mM), DTT (5 mM), 60% (w/v) glycerol and KCl (300 mM) and stored at −25°C. In recent experiments, the purified dimer and other variants were dialyzed against buffer containing Tris–HCl (25 mM; pH 7.5), EDTA (1 mM), DTT (0.1 mM), KCl (300 mM), frozen by dipping in liquid N_2_ (100 μl aliquots in 1.5 ml tubes) and stored at −80°C. The frozen preparation was thawed on ice before use. The concentration of npRecA dimers was determined using *E*^1 M in subunit^_280 nm_ = 21,840.

### Purification of RecA-wt and other variants

*Wild-type* RecA (RecA-wt) was purified as previously described (21,33). The additional information about RecA-wt and the purification of other variants are described in Supplementary Data.

### Gel-filtration profile

Gel-filtration is described in Supplementary Data.

### Dynamic light-scattering

Dynamic light-scattering by the npRecA dimer was measured under the conditions described in Table 1 using the Zetasizer Nano S (Red badge) ZEN1600 instrument and Zetasizer software (Malvern Instruments Ltd, Worcestershire, UK). We measured each sample at least 10 times.

**Table 1.** Dynamic light-scattering analysis of the npRecA dimer with or without ATPγS

	Diameter (nm)^a^	Estimated molecular mass (kDa)^b^	
npRecA dimer alone	7.68 (± 0.28)^c^	78.6 (± 6.8)	(*N* = 17)^d^
npRecA dimer + ATPγS	7.08 (± 0.28)	65.0 (± 6.0)	(*N* = 13)

^a^The npRecA dimer (70 kDa, 0.5 mg/ml, 14 μM as RecA subunit) was mixed with/without ATPγS (0.02 mM) and MgCl_2_ (2 mM) in buffer containing 25 mM Tris–HCl pH 7.5, 1 mM EDTA, 0.1 mM DTT and 150 mM KCl. DLS was measured at 25°C.

^b^Estimated molecular mass was calculated from the diameter, assuming that the protein was spherical. Estimated molecular mass of control proteins (conalbumin 75 kDa, ovalbumin 44 kDa, carbonic anhydrase 29 kDa) were 74.3 ± 3.5, 52.6 ± 3.1 and 32.4 ± 2.3 kDa, respectively. Diameters were 7.50 ± 0.15, 6.47 ± 0.16 and 5.26 ± 0.16 nm, respectively.

^c^In parenthesis, standard deviation.

^d^ *N*, number of analyses.

### Electrophoretic mobility-shift assay for ssDNA-binding activity of RecA variants

The details of the assay are as described previously (21), and in Supplementary Data.

### DFM (Scanning-probe microscopy in dynamic force mode) observation of the npRecA dimer–DNA complex

RecA (final 0.2 μM), npRecA dimer (final 0.2 μM) or npRecA monomer (final 0.8 μM) was added to a reaction buffer (final 20 μl) containing linear dsDNA (2.0 μM; pUC119 digested with *Hin*dIII), ATPγS (0.5 mM), MgCl_2_ (10 mM), Tris–HCl (30 mM; pH7.5) and DTT (1 mM) and incubated for 30 min at 37°C. After 3-fold (RecA and npRecA dimer) or 12-fold (npRecA monomer) dilution with the reaction buffer without RecA and DNA, the reaction mixture (20 μl) was dropped onto a mica surface (1 cm × 1 cm) and left for 15 min at 37°C. After the reaction buffer was removed from the mica, fresh reaction buffer (50 μl) containing glutaraldehyde (0.2%) without RecA and DNA was dropped onto the mica surface and left for 10 min at 37°C. The mica surface was washed five times with water (100 μl) filtered through a filter (Φ 0.22 μm) and dried with an air-blower. The RecA variant-dsDNA complexes were observed by DFM with an SI-DF20S cantilever, which has a tip with a 2–5 nm curvature, with Nano Navi S-image (Hitachi High-Technologies Corporation).

### Assay for ATP/dATP hydrolysis by RecA

Adenosine triphosphatase (ATPase) activities were assayed, as described previously (21,33). The details are described in Supplementary Data.

### D-loop assay for homologous joint formation and branch migration by RecA

Homologous joint formation and branch migration were analyzed by D-loop assay, as previously described (6,21). It should be noted that the branch migration is observed by the dissociation of D-loops formed at the initial phase of the reaction (see Supplementary Data). Briefly, [^33^P]oligo-ssDNA (0.05 μM) and the indicated amounts of RecA variant were incubated for 5–15 min at 37°C in a standard buffer containing dATP (1.3 mM; with an ATP regeneration system) or ATPγS (1.3 mM) instead of ATP, after which joint formation was initiated by the addition of homologous negatively supercoiled dsDNA (18 μM). After incubation at 37°C for the indicated times, the reaction was terminated, the DNA products were fractionated by agarose gel electrophoresis and the ^33^P-signals were analyzed using a BAS-2500 image analyzer. The details of the D-loop assay as well as the principles of branch migration observation by D-loop assay are described in Supplementary Data.

### Fluorometric ssDNA unstacking assay using 6-MI oligo ssDNA

The fluorescence of 6-methylisoxanthopterin (6-MI) oligo ssDNA (3.0 μM) was measured at 25°C, with excitation at 340 nm. The 6-MI oligo ssDNA in Tris–HCl buffer (25 mM; pH 7.5) containing MgCl_2_ (10 mM) and DTT (1 mM) was incubated at 25°C in fluorescence cuvettes. The RecA variants were titrated into the cuvettes to the indicated concentrations in the presence or absence of ATPγS (0.04 mM). At each concentration of RecA variant, measurements were performed after incubation at 25°C and stirring for 3 min.

## RESULTS

### NpRecA monomers and npRecA dimers do not polymerize in solution

**Figure 2.**
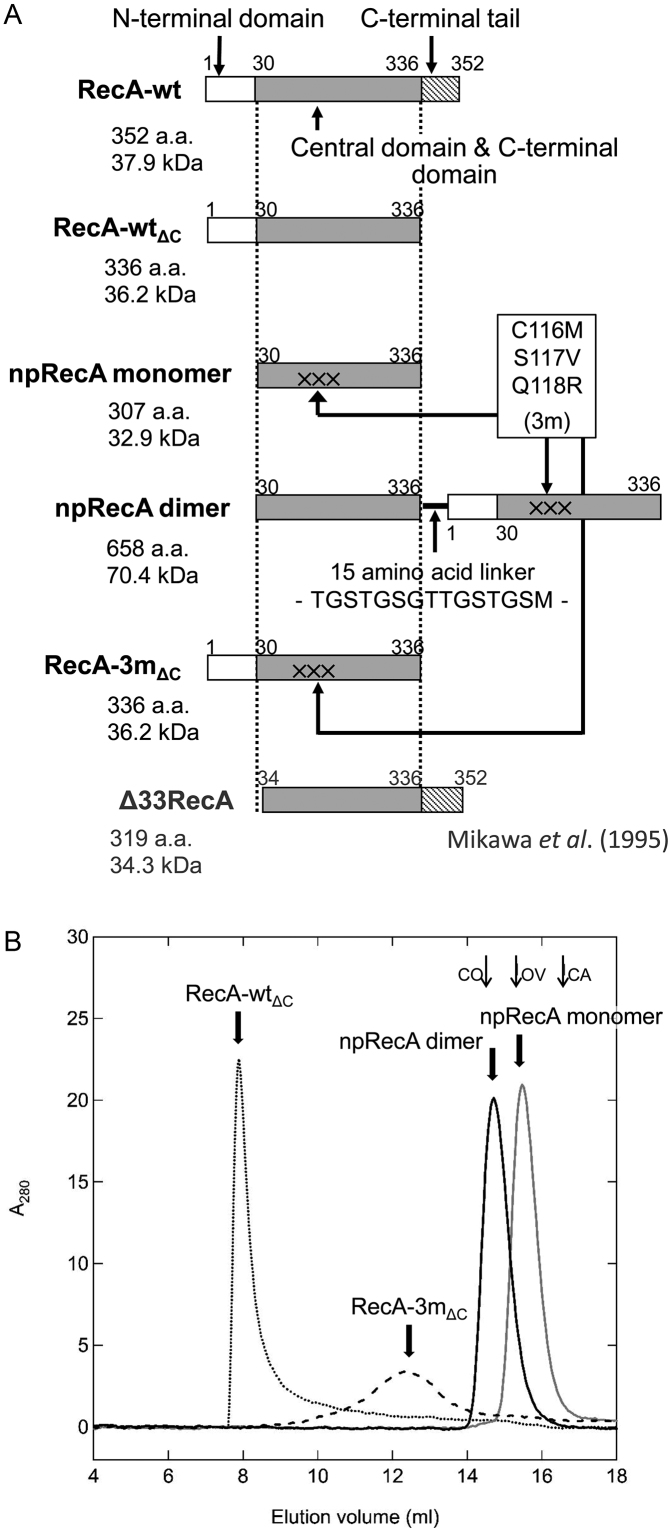
Design of RecA variants and the absence of polymer formation in free forms of npRecA variants. (**A**) Domain structure of RecA variants.The RecA and RecA variants are: RecA-wt (*wild-type* RecA), RecA-wt_ΔC_ (the RecA variant with the C-terminal 16 amino-acid deletion that is part of the C-terminal 25 amino-acid-disordered region; 34), RecA-3m_ΔC_ (RecA-wt_ΔC_ variant which bears the 3m three-residue replacement [C116M, S117V, Q118R]), the nonpolymerizing (np) RecA monomer, the npRecA dimer (the nonpolymerizing head-to-tail-fused RecA dimer) and Δ33RecA (truncated RecA lacking the N-terminal 33 amino-acid residues; Mikawa *et al*. (1995), see ref. 35). The concentration of npRecA dimers was expressed as the concentration of RecA subunits. Thus, 1 mol of the npRecA dimer is expressed as 2 mols in this paper. (**B**) Gel-filtration profiles of the RecA variants. RecA variants (10 μM 100 μl) were analyzed by chromatography on a Superdex200 10/300 GL column. Molecular-sized markers: conalbumin (CO, 75 kDa), ovalbumin (OV, 44 kDa) and carbonic anhydrase (CA, 29 kDa).

RecA has three well-structured domains, the N-terminal, core and C-terminal domains. The major inter-protomer interfaces of RecA in the RecA filaments, either in the presence of both ATP and DNA or in their absence, include the first α-helix A (D3 to F21) and β-strand 0 (I26 to R28) in the N-terminal domain, and the β-strand 3 (from L114 or L115 to S117 or Q118) in the core domain (18,34). Truncated RecA lacking the N-terminal region (Δ33RecA; Figure 2A) still forms multimers at high concentrations (35). This indicates that additional mutations are required to completely abolish the multimer-forming or filament-forming activity of RecA. One amino-acid residue in the interface on the core domain, S117, causes mild recombination deficiency of *E. coli* when replaced (36). Three amino-acid substitutions, C116M, S117V and Q118R (3m substitution) were included in the C-terminal unit of the RecA oligomer constructs for co-crystallization with DNA (18). By referring to the constructs used in this co-crystallization, we designed an npRecA monomer and a covalently linked head-to-tail npRecA dimer (Figure 2A).

The npRecA monomer had the N-terminal 29-residue truncation and the 3m substitution in the core domain (Figure 2A). The npRecA dimer consisted of an N-terminal RecA subunit with the 29-residue truncated N-terminus and a C-terminal RecA subunit with the 3m substitution, connected in tandem by a 15-residue linker. Note that the npRecA dimer retained the intact inter-protomer interfaces between the RecA subunits. We prepared a RecA-wt_ΔC_ (see below) variant with the 3m substitution (RecA-3m_ΔC_) as a control to determine the effects of the 3m substitution (Figure 2A).

All of the RecA subunits or protomers of the RecA variants have the C-terminal 16-amino-acid deletion (Phe-337 to the C-terminal Phe-352; Figure 2A). We left one acidic-amino acid, Asp-336, from the RecA C-terminal acidic residue-rich unstructured tail, at the C-terminus, to decrease the effect of the deletion on strand exchange (see (37)). The purified preparations of the npRecA dimer and the npRecA monomer each contained a single major species of the expected size (Supplementary Figure S1A). We confirmed the proper folding of the prepared RecA variants by measuring their circular dichroism (CD) spectra (Supplementary Figure S1B). The *wild-type* RecA variant with the C-terminal 16-amino-acid deletion (RecA-wt_ΔC_) was shown to have activity levels for homologous joint formation and branch migration similar to those of the *wild-type* RecA (RecA-wt), especially in the presence of dATP (Supplementary Figure S2), as detected by D-loop assay (see Supplementary Methods; Supplementary Figure S2A and B), and ssDNA-dependent dATP/ATP hydrolysis (Supplementary Figure S2C). We therefore used RecA-wt as well as RecA-wt_ΔC_ as positive controls.

We subjected the npRecA monomer and the npRecA dimer to chromatography (10 μM, ∼5–10-fold higher than the concentrations used in the biochemical assays) on a Superdex200 10/300 GL column and analyzed their gel-filtration behavior. Like RecA-wt (e.g. (35)), RecA-wt_ΔC_ showed self-polymerization and behaved as a large multimer during gel filtration (Figure 2B). The RecA-3m_ΔC_ variant showed a broad and symmetric peak at a position for a larger protein than that of the npRecA dimer (Figure 2B), suggesting that RecA-3m_ΔC_ forms oligomers but is partially defective in multimerization. The npRecA monomer (36.2 kDa) eluted from the column slightly after the 44 kDa marker protein, and the npRecA dimer (70.4 kDa) eluted slightly after the 75 kDa marker protein (Figure 2B). Thus, the npRecA monomer and the npRecA dimer behaved as monomer and dimer, respectively, during gel filtration.

We further analyzed the molecular size of the npRecA dimer in solution using dynamic light-scattering in the presence and absence of ATPγS, an unhydrolyzable ATP analogue. ATP binds to RecA in the clefts of two adjacent RecA protomers, and thus, ATPγS may bridge two npRecA dimers. We calculated the diameter of the npRecA dimer from the dynamic light-scattering data, and estimated its molecular mass from the diameter (Table 1 and Supplementary Figure S3), assuming that the dimer is spherical. The calculated diameters have a symmetrical distribution with a single peak in both the absence or presence of ATPγS (Supplementary Figures S3A and B). The estimated molecular masses shown in Table 1 indicate that the npRecA dimer behaves as a dimer in solution in the presence or absence of ATPγS. A slight decrease in diameter would reflect the conformational change of the npRecA dimer upon ATPγS-binding.

### The npRecA dimer is active in DNA binding

**Figure 3.**
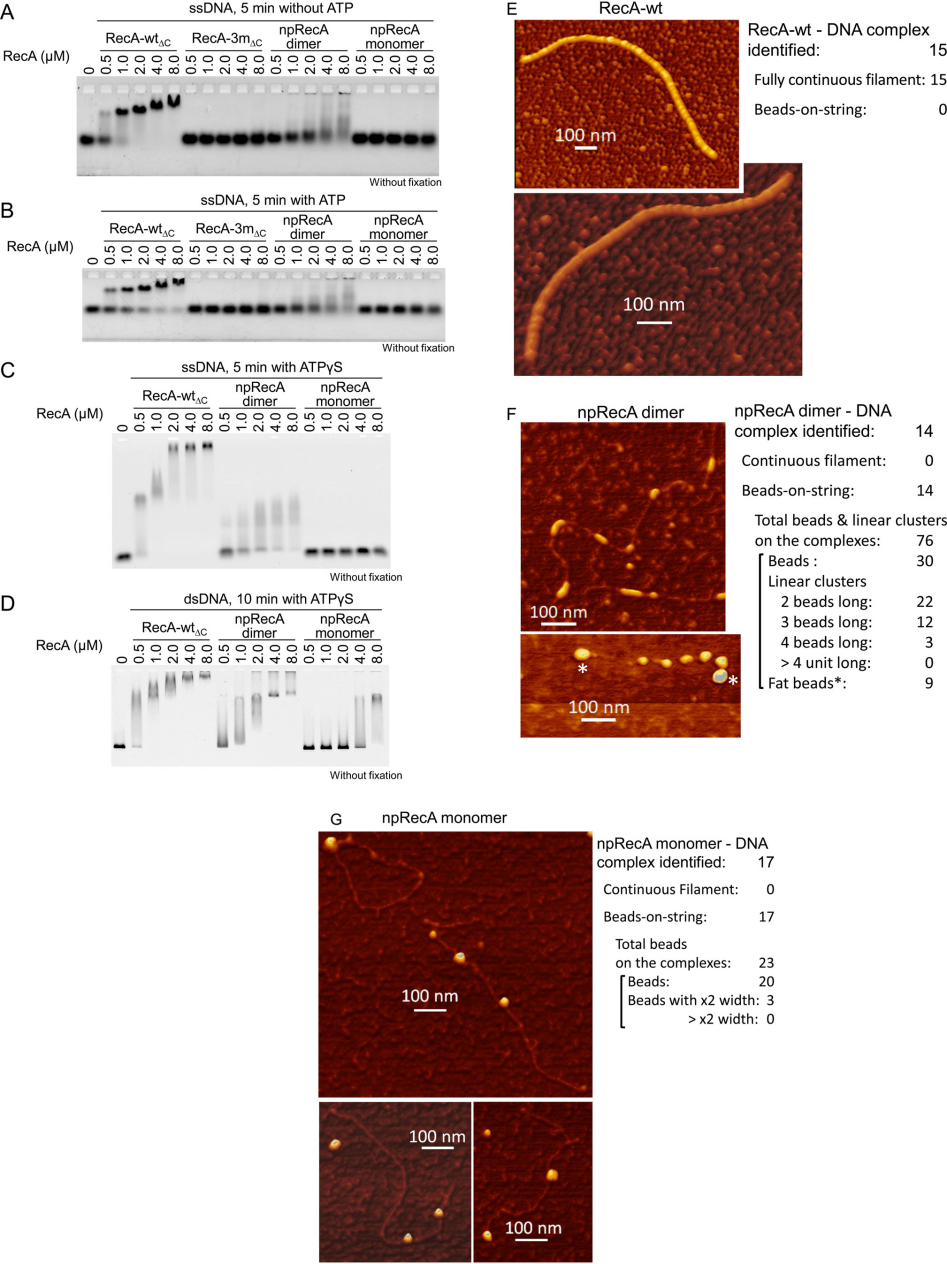
NpRecA dimers binds to DNA but does not form filaments. (**A, B** and **C**) ssDNA-binding activities of npRecA dimers detected by electrophoretic mobility-shift assay. FAM-labeled 90-mer ssDNA (4.5 μM) was incubated in a standard reaction buffer with the indicated amounts of RecA variants in the absence of nucleotide cofactor (**A**), the presence of ATP (1.3 mM) (**B**), or ATPγS (1.3 mM) (**C**). DNA samples without fixation were electrophoresed through an agarose gel. FAM-labeled DNA was detected using a biomolecular imager. (**D**) dsDNA-binding activities of npRecA dimers and npRecA monomers detected by electrophoretic mobility-shift assay. Unlabeled pUC119 dsDNA linearized by *Hin*dIII (9.0 μM) was incubated as in A to C in the presence of ATPγS (1.3 mM). After electrophoresis, dsDNA was detected by ethidium bromide-staining using a biomolecular imager. (**E** and **F**) NpRecA dimers form beads-on-string structures instead of filaments upon DNA binding. RecA-wt (**E**) and npRecA dimers (**F**) at 0.2 μM (RecA subunit) were allowed to form a complex with linearized pUC119 dsDNA (2.0 μM) in the presence of ATPγS (0.5 mM) and MgCl_2_ (10 mM). After 3-fold dilution, the complexes were fixed with glutaraldehyde (0.2%) on a mica surface and visualized by DFM (a scanning-probe microscope in dynamic force mode). (**G**) Beads-on-string structures formed by the npRecA monomer bound to dsDNA. The npRecA monomer at 0.8 μM was allowed to form a complex with dsDNA as described above. After 12-fold dilution, the complexes were visualized by DFM as described above.

In homologous joint formation, RecA first binds to ssDNA. Therefore, we tested the RecA variants for their ssDNA-binding ability via a electrophoretic mobility-shift assay, using FAM-labeled 90-mer ssDNA (OL2). RecA bound cooperatively to ssDNA to form nucleoprotein filaments, and filament formation stabilized the binding of RecA and ssDNA. RecA-3m_ΔC_ retained its oligomer-forming activity (Figure 2B) but was defective in ssDNA binding (Figure 3A and B). Likewise, the npRecA monomer was completely defective in ssDNA binding (Figure 3A–C). Although more proteins were required and the shift of signals was smaller than with RecA-wt_ΔC_, the npRecA dimer was active in binding to ssDNA in the presence or absence of ATP or ATPγS (Figure 3A–C).

The quick binding of RecA to dsDNA requires the presence of ssDNA, but in the presence of ATPγS, RecA binds dsDNA in the absence of ssDNA (17), and forms nucleoprotein filaments (12). As expected, the npRecA dimer bound to dsDNA in the presence of ATPγS, though more proteins were required than with RecA-wt (Figure 3D). Unexpectedly, we also found that the npRecA monomer bound to dsDNA under these conditions (Figure 3D) although many more proteins were required than with the dimer. Since the npRecA monomer did not bind to ssDNA at all, as described above, this result indicates that the npRecA monomer has a dsDNA-specific binding activity.

### The npRecA dimer does not form continuous filaments on DNA

As described above, the npRecA dimer does not polymerize in a DNA-free form in the absence or presence of nucleotide-cofactor (Figure 2B and Table 1). But this does not exclude the possibility that, upon binding to DNA in the presence of ATP or its analogue, the npRecA dimer forms nucleoprotein filaments by residual weak interactions between npRecA dimers. We examined this possibility using DFM. Since free ssDNA appears as collapsed clumps (e.g. see (22)), which makes it impossible to distinguish between a discretely bound form and a continuous filament form of an RecA variant, we employed dsDNA for this analysis. In the absence of ssDNA, RecA-wt binds to dsDNA in the presence of an unhydrolyzable ATP analogue such as ATPγS, and forms a continuous filament along the dsDNA, as described above. It is known that ssDNA-bound RecA-wt and dsDNA-bound RecA-wt form identical filamentous structures (38,39), even at atomic resolution (18).

We incubated npRecA dimers and linear dsDNA in the presence of ATPγS, then compared the resulting complex with RecA-wt. Since interaction with the scanning tip during atomic-force microscopic analysis can cause dissociation of RecA-wt from DNA (40), we used DFM (41) and glutaraldehyde fixation of the RecA-DNA complex. The fixation did not affect the structure of the RecA-DNA complex (42). As reported previously, RecA-wt formed a continuous filament (Figure 3E), whereas the npRecA dimer did not form a continuous filament at all (Figure 3F). Instead, the npRecA dimer formed beads-on-string structures (Figure 3F). The majority (68%) of the proteins detected (*N* = 76) on the strings were beads and linear clusters of two beads long, both of which have the same width. The width is smaller than that of the RecA filament (Figure 3E and F).

Although the lateral resolution was insufficient to visualize the details of the RecA dimer, the vertical (z-axis) resolution was much higher in the DFM analysis (41). The height of the RecA-wt filaments was 4.9 (±1.0) nm (in parenthesis, standard deviation (SD), number of analysis (*N*) = 8), almost the same size (3.2–4.2 nm) as those reported previously (e.g. (42)), while the height of the beads and linear clusters of the npRecA dimer was 1.9 (±0.2) nm (*N* = 10). Thus the height (z-axis) of the beads and linear clusters of the npRecA dimer was one third to half that of the filament formed by RecA-wt. From these observations, we conclude that, unlike RecA-wt, the npRecA dimer is unable to form filaments upon binding to DNA.

Since the electrophoretic mobility-shift assay showed that the npRecA monomer binds to dsDNA, we used DFM to analyze the complex formed by the npRecA monomer and dsDNA. All the complexes identified were beads-on-string, of which 87% are beads with almost same width as the npRecA dimer beads (Figure 3G). The beads of the npRecA monomer were spherical, and have almost same width as the npRecA dimer beads, which were slightly elongated along the DNA (Figure 3G and F). These images reflect the monomer and dimer molecules bound to DNA.

When we replaced dsDNA with circular phage ssDNA for this DFM analysis, RecA-wt formed continuous filaments, the appearance of which was indistinguishable from those formed with dsDNA (Supplementary Figures S4A, C and Figure 3E), as reported previously (22). The npRecA dimer-ssDNA complex formed squat columns of uniform size (especially the diameter), which were several times larger than the beads on dsDNA (Supplementary Figures S4B, D and Figure 3F). As we expected for the above reason, we observed no visible ssDNA attached to the columns in the DFM images (Figure S4B). It is likely that the squat columns represent npRecA dimer-ssDNA complexes condensed during the drying step of the specimen preparation. These results also indicate that the npRecA dimer is unable to form continuous filaments upon binding to ssDNA.

### The npRecA dimer catalyzes ATP/dATP hydrolysis

**Figure 4.**
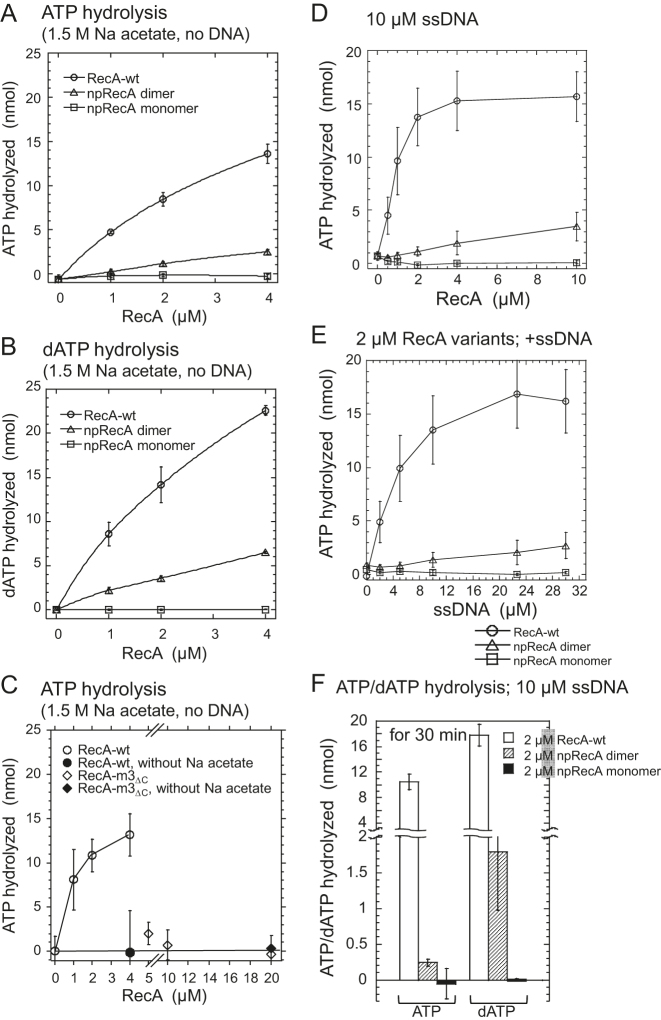
ATP/dATP hydrolyzing activities of RecA variants. All incubation was carried out in a standard reaction buffer (20 μl). (**A**–**C**) DNA-independent ATP/dATP hydrolysis. The amount of ATP (A and C) or dATP (B) hydrolyzed during incubation (15 min) in the presence of sodium acetate (1.5 M) was plotted against the indicated amounts of RecA variants. The white symbols represent a complete system for DNA-independent hydrolysis of ATP or dATP by the RecA variants; the black symbols represent the absence of sodium acetate. Circles represent RecA-wt; triangles represent the npRecA dimer; squares represent the npRecA monomer; and diamonds represent RecA-3m_ΔC_. Each value is the average of data from at least three independent experiments. (**D** and **E**) ssDNA-dependent ATP hydrolysis with various amounts of RecA variants and ssDNA. ATP hydrolysis was carried out by a 30 min incubation at 37°C with circular ssDNA (10 μM) and the indicated amounts of RecA variants (**D**), or with RecA variants (2.0 μM) and the indicated amounts of circular ssDNA (**E**). Circles represent RecA-wt; triangles represent the npRecA dimer; squares represent the npRecA monomer. Values represent the average of data from at least three independent experiments. (**F**) ssDNA-dependent ATP/dATP hydrolysis. The amount of ATP/dATP hydrolyzed during incubation (30 min) in the presence of circular ssDNA (10 μM) and RecA-wt or the RecA variants (2.0 μM) was plotted. We chose the concentrations of RecA variants and ssDNA within the limits of those in which ATP hydrolysis linearly depended in the concentrations of RecA variants (D) and ssDNA (E). In contrast with (A) to (E), the observed amounts of ADP and dADP in the controls without DNA were subtracted from the values obtained in the presence of DNA in each experiment. The observed values of the control without DNA were 0.8 and 0.2 nmol for ADP and dADP, respectively, for RecA-wt, and 0.12 and 0.14 nmol for ADP and dADP, respectively, for the npRecA dimer. White squares represent RecA-wt, diagonally striped squares represent the npRecA dimer and black squares represent the npRecA monomer. Each value represents the average of data from at least three independent experiments.

RecA has an ssDNA-dependent ATP-hydrolyzing activity (11). As in homologous joint formation, ssDNA-dependent ATP hydrolysis is also promoted on the active (or extended) RecA filament (see (18)). Since the npRecA dimer did not form filaments on DNA, we questioned whether the npRecA dimer was active in ATP hydrolysis. However, because of the reduced ssDNA binding (Figure 3A and B), we were unable to estimate the contribution of ATP hydrolyzing defects from a reduced level of ssDNA-dependent ATP hydrolysis. We chose to examine the DNA-independent ATPase activities of the RecA variants. A high concentration of salt can take the place of DNA in ATP hydrolysis by RecA (43). We thus measured the extent of ATP/dATP hydrolysis by the npRecA dimer compared with the RecA controls, in the presence of sodium acetate (1.5 M) instead of DNA. It has been reported that dATP is a more effective nucleotide cofactor than ATP for the molecular activities of *E. coli* RecA (44,45). In agreement with these reports, we observed that more dATP was hydrolyzed by RecA-wt than ATP in the presence of sodium acetate (Figure 4A and B).

Since each ATPase active site consists of the residues of the two adjacent RecA protomers, the absence of any interaction between the npRecA dimers will result in a halving of the number of active ATPase sites per RecA protomer (or subunit), compared with RecA-wt, and thus in a reduction in ATPase activity to half that of RecA-wt. In fact, RecA variants defective in the inter-protomer interface, the npRecA monomer and RecA-3m_ΔC_, did not exhibit any detectable ATP hydrolysis in the presence of sodium acetate (1.5 M; Figure 4A–C). Under these conditions, the npRecA dimer hydrolyzed ATP and dATP at a level between one fifth and one third, respectively, that of RecA-wt, or two fifths and two thirds, respectively, per an ATPase site of RecA-wt (Figure 4A and B).

When we examined the ssDNA-dependent ATPase activities of the RecA variants, we found that the npRecA dimers showed weak but detectable activity, whereas the npRecA monomer did not show any detectable activity (Figure 4D–F). When we compared npRecA dimer with RecA-wt as for ssDNA-dependent ATP/dATP hydrolysis, we found that the npRecA dimer hydrolyzed much more dATP than ATP relative to their hydrolysis by RecA-wt (Figure 4F). This suggests that the npRecA dimer is more active in the presence of dATP than in the presence of ATP. We therefore decided to use dATP in our experiments on homologous joint formation.

### The npRecA dimer, but not the npRecA monomer, catalyzes homologous joint formation

As described in the Introduction, using a D-loop assay consisting of oligo ssDNA and homologous negatively supercoiled dsDNA (pBluescript SK(-)), we were able to test the npRecA dimer and the npRecA monomer for homologous joint-formation activity. We used OL2 oligo ssDNA (90-mer ssDNA) for the oligo ssDNA, as it has a minimal secondary structure (see (21)). This allowed us to assess the homologous joint-formation activities of the RecA variants, even if they were defective in the unfolding of ssDNA secondary structures.

**Figure 5.**
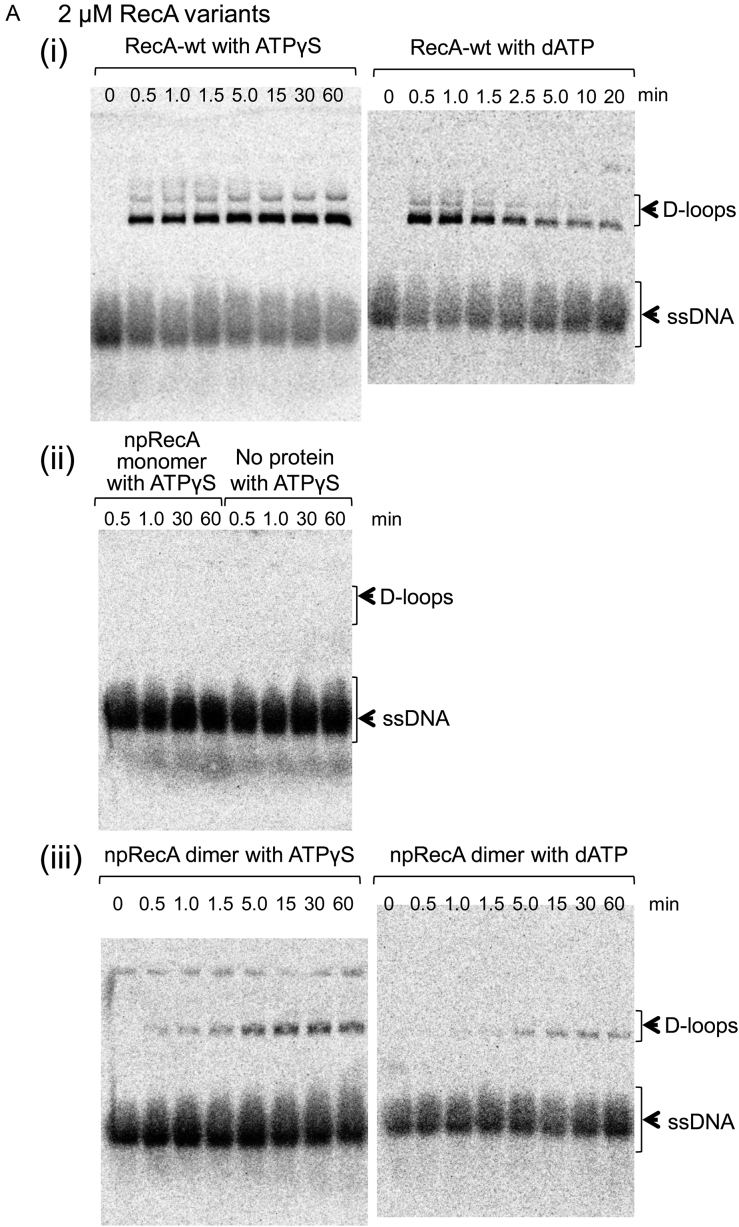

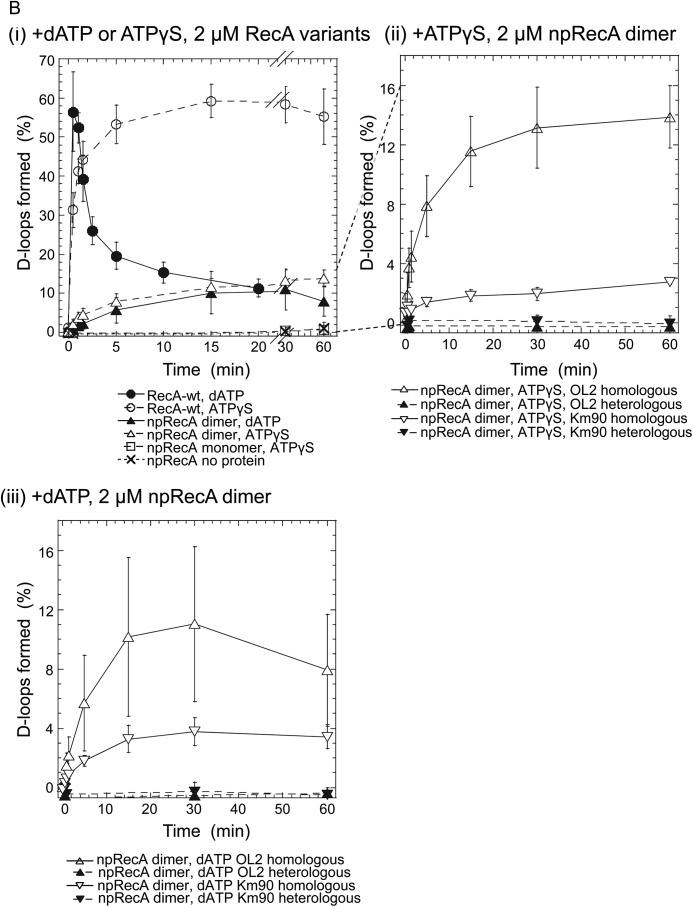
Homologous joint formation by the npRecA dimer as shown by D-loop assay. 90-mer [^33^P] oligo-ssDNA (0.05 μM) and RecA variant (2 μM) were incubated in a standard reaction buffer at 37°C in the presence of ATPγS or dATP (1.3 mM) for 5–15 min, following which homologous negatively supercoiled dsDNA (18 μM) was added to initiate the reaction. Mixtures were incubated at 37°C for the indicated times. (**A**) Formation of D-loops as represented by gel profile. OL2 ssDNA and homologous pBluescript SK(–) negatively supercoiled dsDNA were incubated with (i) RecA-wt, (ii) the npRecA monomer or no protein added and (iii) the npRecA dimer for the indicated times. (**B**) Quantitative representation of D-loop formation. (i) D-loop formation by RecA variants in the presence of ATPγS or dATP. OL2 ssDNA and homologous pBluescript SK(–) negatively supercoiled dsDNA were incubated with the indicated RecA variants. White symbols represent the presence of ATPγS; black symbols represent the presence of dATP. Circles represent RecA-wt; triangles represent the npRecA dimer; squares represent the npRecA monomer; the X represents without protein. (ii) D-loop formation by the npRecA dimer in the presence of ATPγS. (iii) D-loop formation by the npRecA dimer in the presence of dATP. In (ii) and (iii), the white symbols represent homologous combination of ssDNA and negatively supercoiled dsDNA and the black symbols represent heterologous combination of ssDNA and negatively supercoiled dsDNA. Triangles represent the npRecA dimer, OL2 ssDNA and homologous or heterologous dsDNA and inverted triangles represent the npRecA dimer, Km90 ssDNA and homologous or heterologous ssDNA. OL2 ssDNA is homologous to pBluescript SK(–) dsDNA, but heterologous to pKF18 dsDNA. Km90 ssDNA is homologous to pKF18 dsDNA, but heterologous to pBluescript SK(–) dsDNA. Each value represents the average of data from at least three independent experiments. The values for the npRecA dimer, OL2 ssDNA and pBluescript SK(–) dsDNA shown in (ii) and (iii) are identical to those shown in (i) for the presence of ATPγS and dATP, respectively.

In the presence of ATPγS, RecA-wt formed D-loops with more than one third of the ssDNA after 1.5 min incubation. After 15 min, joint formation had increased to more than half the ssDNA (see Materials and Methods; Figure 5A(i) left panel and B(i)). As expected from the absence of the filament formation that stabilizes nascent joint (see Introduction), the yield was reduced to a quarter the amount, compared to RecA-wt, but we did find that the npRecA dimer catalyzed D-loop formation in the presence of ATPγS (Figure 5A(iii) and B(i)). By contrast, and as expected because of the absence of ssDNA binding, the npRecA monomer did not catalyze D-loop formation at all under these conditions (Figure 5A(ii) and B(i)).

To confirm that the signal detected was sequence-homology-dependent, we replaced the homologous dsDNA (pBluescript SK(–)) with a heterologous dsDNA (pKF18). No signal for D-loop formation was detected (Figure 5B(ii)). When we replaced the OL2 oligo ssDNA with the Km90 ssDNA (90-mer), we detected D-loop formation by the npRecA dimer with homologous dsDNA (pKF18 dsDNA), but not with heterologous pBluescript SK(–) dsDNA (Figure 5B(ii)). Yield was decreased by a quarter with the Km90 ssDNA, probably because of the difference in their secondary structure. These results indicate that the signals detected by the D-loop assay were truly sequence-homology-dependent events (i.e. indicative of homologous joint formation), and excluded any other events.

We next tested dATP, which supports homologous joint formation and subsequent branch migration by RecA-wt (Figure 5A(i) and B(i), and Supplementary Figure S2A). As seen in these figures, RecA-wt and RecA-wt_ΔC_ formed D-loops in the presence of dATP within 30 s, and rapidly dissociated the joints by branch migration (see D-loop assay for branch migration in Methods). In the presence of dATP, the npRecA dimer catalyzed D-loop formation with either OL2 oligo ssDNA or Km90 ssDNA (specifically, with their homologous dsDNA) with a rapidity and yield similar to that seen in the presence of ATPγS (Figure 5A and B). Dissociation of the D-loops after formation was not significant with the npRecA dimer (Figure 5B(i and iii)).

These results indicate that the npRecA dimer, but not the npRecA monomer, catalyzes homologous joint formation in the absence of filament-forming activity.

### Homologous joint formation examined by strand-exchange assay

A strand-exchange assay using circular ssDNA and homologous linear dsDNA is often used to study homologous joint formation catalyzed by RecA and Rad51. This assay enables us to discriminate three successive phases of homologous joint formation: presynaptic filament formation (39), homologous pairing (sometimes called ‘synapsis’), and branch migration (15,16). For homologous joint formation to be detected by a strand-exchange assay, the unfolding of ssDNA by continuous filament formation around the ssDNA in presynaptic phase is required (39,46,47). As expected from the absence of filament formation by the npRecA dimer, neither the npRecA dimer nor the npRecA monomer formed detectable homologous joints under these conditions (Supplementary Figure S5).

### The npRecA dimer is active in base unstacking

The fluorescence of etheno-modified ssDNA (ϵssDNA), in which adenine was etheno-modified, was increased several fold by RecA binding, and to a further extent by the addition of ATP or its unhydrolyzable analogue, ATPγS (Supplementary Figure S6; 48,49). This increase is explained by the quenching of the fluorescence by base stacking in free ssDNA, and by base unstacking due to RecA binding to the DNA (49). ATP- and ATPγS-dependent increases in fluorescence correlate with the extension of RecA-ssDNA filaments associated with the activation of homologous joint formation (39,50,51). Thus, fluorescence enhancement of ϵssDNA by the addition of ATP or ATPγS in the presence of RecA is an indicator for ATP- or ATPγS-dependent activation of the RecA-ssDNA complex for homologous joint formation. Fluorescence enhancement of ϵssDNA was measured as the fluorescence of a sample minus the fluorescence of the ϵssDNA control without RecA. As with RecA-wt, an increase in the fluorescence enhancement of ϵssDNA by the addition of ATPγS was observed in the presence of the npRecA dimer (Supplementary Figure S6B). However, this assay showed the presence of a nonspecific interaction between the npRecA monomer and ϵssDNA (Supplementary Figure S6B).

**Figure 6.**
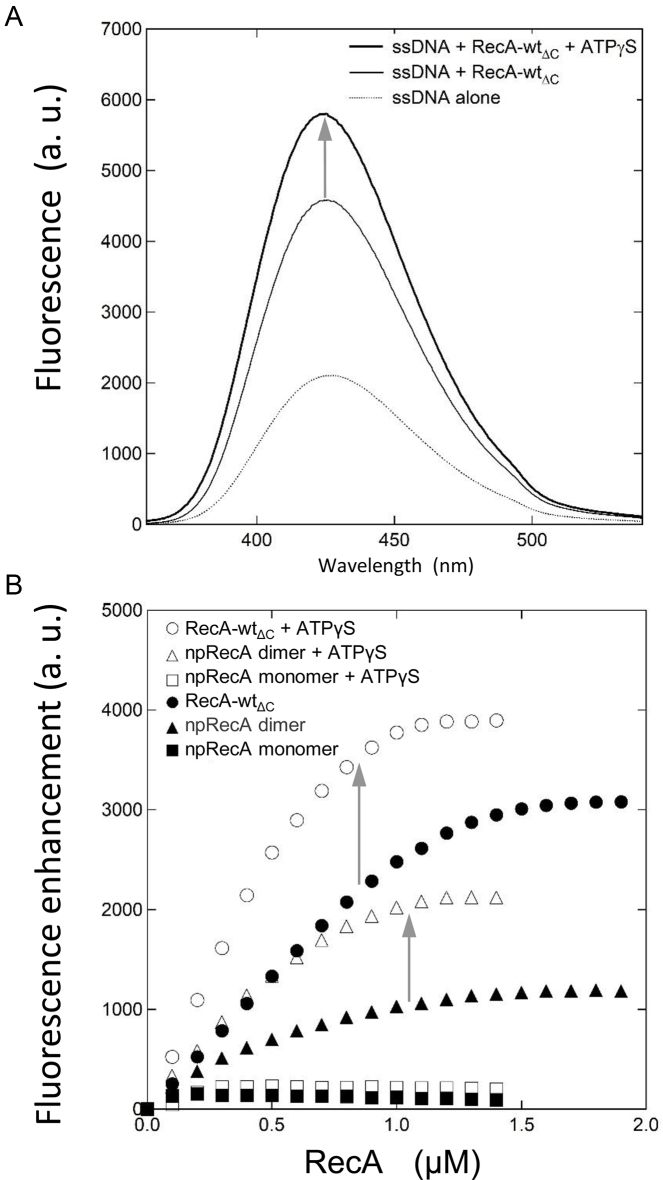
Base unstacking of ssDNA by RecA variants. (**A**) Fluorescence-emission spectra of 6-MI oligo ssDNA in the presence or absence of RecA-wt_ΔC_ and ATPγS. Fluorescence of 6-MI oligo ssDNA (3.0 μM) was measured at 25°C in the presence of RecA-wt_ΔC_ (1.0 μM) in the absence or presence of ATPγS (0.04 mM). Fluorescence is expressed in arbitrary units (a.u.). (**B**) Relief of fluorescence quenching of 6-MI oligo ssDNA by RecA variants in the presence or absence of ATPγS. Fluorescence at 425 nm was measured at 25°C in the presence of 6-MI labeled oligo-ssDNA (3.0 μM) and the indicated amounts of RecA variants, with or without ATPγS. Fluorescence enhancement of 6-MI oligo ssDNA was defined as the fluorescence of a sample minus the fluorescence of the 6-MI oligo ssDNA control without RecA. White symbols represent the presence of ATPγS (0.04 mM) and black symbols represent the absence of ATPγS. Circles represent RecA-wt_ΔC_, triangles represent the npRecA dimer and squares represent the npRecA monomer.

We eliminated the false signals from non-functional interactions between DNA and protein and strengthened the results by introducing a new fluorescence assay using 6-methylisoxanthopterin (6-MI)-labeled 42 mer ssDNA (6-MI oligo ssDNA) with a defined sequence. While etheno-modified adenines do not form base pairs with thymine bases on the complementary strand, 6-MI modification does not affect base pairing with the complementary strand (52). The fluorescence emitted from the 6-MI oligo ssDNA was increased by the addition of either RecA (RecA-wt_ΔC_) or the npRecA dimer in the absence of a nucleotide cofactor, and was further increased by the addition of ATPγS (Figure 6A and B). Unlike ϵssDNA, the addition of the npRecA monomer did not increase fluorescence (Figure 6B), indicating that the increase in the fluorescence of 6-MI-labeled ssDNA is specific to the functional binding of RecA-wt and the npRecA dimer to ssDNA.

These results indicate that, as with RecA-wt (or RecA-wt_ΔC_), ssDNA is extended and its bases are unstacked by the binding of the npRecA dimer, but not the npRecA monomer, and that unstacking is further enhanced by the addition of ATPγS.

## DISCUSSION

Since the discovery of unique right-handed filamentous structures formed by RecA with dsDNA or ssDNA (12,13,22), it has been generally believed that the filamentous structure that forms around ssDNA consisting of RecA/Rad51 is essential to search and recognition of sequence homology with dsDNA (53,54). The major finding of this study is that, contrary to this accepted notion, the npRecA dimer, but not the npRecA monomer, catalyzes D-loop formation (Figure 5) without filament formation (Figure 3F and the discussion described below). We therefore conclude that the filament formation is not essential to sequence-homology recognition and pairing for homologous joint formation by RecA, and that a head-to-tail-dimer form of RecA is required and sufficient for RecA-catalyzed sequence-homology recognition and pairing.

This is the first study that shows directly that RecA dimer catalyzes homologous joint formation without forming filaments. Forget *et al*. (55) reported that RecA dimer is a functional unit for assembly of nucleoprotein filaments (not for homologous joint formation) from the experiments using a fused dimer of RecA with two amino-acid substitutions (K6A and R28A) within the N-terminal domain of the N-terminal RecA subunit, connected by a five-residue linker to the N-terminus of the C-terminal *wild-type* RecA subunit. This fused RecA dimer formed protein-ssDNA filaments, and was fully active *in vitro* and *in vivo*, compared with RecA-wt (55). Sharma *et al*. (56) claimed that a RecA complex containing 6-nucleotide-long ssDNA can specifically bind to the homologous region of dsDNA, and assume from this result that two RecA protomers can recognize homologous sequence. In the presence of ATPγS (which Sharma *et al*. used), RecA was shown to form nucleoprotein filaments containing tandemly juxtaposed ssDNA molecules, in which the 3′ terminus was juxtaposed with the 5′ terminus of another fragment (57). The sequence-homology recognition shown by Sharma *et al*. can be explained by the possible contribution of a RecA filament consisting of several RecA protomers bound to juxtaposed 6-nucleotide-long ssDNA-oligomers.

The visualization of the npRecA dimer-DNA complex by DFM (Figure 3F and Supplementary Figure S4B) clearly shows that, unlike RecA-wt (Figure 3E and Supplementary Figure S4A), the npRecA dimer does not form continuous filaments, even after binding to DNA, either ss or ds. Thus, N-terminal truncation and 3m substitution completely prevented the npRecA dimer from forming normal nucleoprotein filaments. The lateral resolution of DFM images is not sufficient to exclude the possibility that the beads and linear clusters observed by DFM are npRecA dimers that form small filaments. However, the following observations exclude the possibility that the interfaces between the npRecA dimers in the linear clusters (and beads if each bead consists of multiple molecules of the dimers) observed in the beads-on-string structure of the RecA dimer mimic the interfaces in active RecA filaments and enable the clusters to behave like active RecA filaments: (i) The vertical resolution in DFM is sufficient to identify variations among protein-DNA complexes (41). DFM analysis showed that the height of the beads and the linear clusters of the npRecA dimer was one third to half that of the filament formed by RecA-wt (Figure 3E and F, and the related description in the Results). This result excludes the possibility that the beads and linear clusters formed by the npRecA dimer include RecA-spiral filament structures. (ii) The npRecA monomer is completely defective in ssDNA binding (Figure 3A and B) and ATP hydrolysis in the presence of sodium acetate (1.5 M; Figure 4A and B) or ssDNA (Figure 4D–F) and does not form filaments upon dsDNA binding (Figure 3G), indicating that N-terminal 29-residue truncation and 3m substitution completely eliminate the physical interaction needed to form an active filament, even under conditions favorable to filament formation (i.e., the presence of sodium acetate (58) or DNA (38,39)).

We thus conclude that the npRecA dimer is active in sequence-homology recognition and pairing (Figure 5) in the absence of filament formation.

As with the secondary DNA-binding site for dsDNA, the ATP-binding site is located between two adjacent RecA protomers, and the binding of ATP analogues changes the spatial configuration of the adjacent protomers in the RecA filament (18,34,59). This structural change induces the extension of bound ssDNA for base unstacking and the activation of the secondary DNA-binding site to bind to dsDNA. We infer that the npRecA dimer was activated in the same way, since it exhibited DNA-dependent- and DNA-independent ATP/dATP hydrolysis (Figure 4) and ATPγS-dependent unstacking of bases in ssDNA (Figure 6 and Supplementary Figure S6). Our findings regarding the dsDNA-specific binding of the npRecA monomer suggest that the inactive state of the secondary DNA-binding site includes at least in part the hiding of the dsDNA binding surface located on one side of the cleft between two adjacent RecA protomers (23–25), as it is covered by the other side of the cleft (i.e. the adjacent protomer), and that activation of dsDNA binding includes the uncovering of the binding surface by moving the adjacent protomer.

One obvious significance of this study is that the npRecA dimer would be an ideal tool to reveal the basic mechanism of homologous joint formation; i.e., sequence-homology recognition and pairing. dsDNA binds to a single and unique secondary DNA-binding site of the npRecA dimer-ssDNA complex, thus the resulting complex has a uniform structure. Each RecA-wt filament has multiple secondary DNA-binding sites along the filament. Thus, the RecA–ssDNA filament–dsDNA complex is polymorphic. This feature makes it difficult to obtain high-resolution structural information about dsDNA at the secondary binding sites of the complex, even with the latest analytical techniques, such as high-resolution cryo-electron microscopy (20) and X-ray crystallography (18).

This study reveals that filament-independent sequence-homology recognition and pairing can be experimentally separated from the filament-dependent process, and then, our findings raise this issue: what are the exact and essential functions of filament formation by RecA/Rad51 in homologous recombination and proliferation? The difference in D-loop formation efficiency between RecA-wt and the npRecA dimer (Figure 5B) indicates that filament formation by RecA contributes to the efficiency of homologous joint formation.

A well known function of presynaptic filament formation is the unfolding of secondary structures of ssDNA (39), but this function can be substituted by single-strand binding protein (SSB; 60). After nascent homologous joints are formed by RecA-wt, the nascent homologous joints are extended to minimum stable joints of about 14/15 bp, in the absence of ATP hydrolysis (29,31). This extension is likely to be a filament function as described in Introduction. The homologous joints are further extended to a length of thousands of base pairs by filament-formation- and ATP-hydrolysis-dependent reactions by RecA (see the Introduction). However, the two structurally related nonfilament-forming proteins, Rad52 (61–63) and β protein (64,65), are known to catalyze D-loop formation in the absence of ATP *in vitro*. Although Rad52 is essential to Rad51-dependent recombination, Rad52 also plays a role in Rad51-independent homologous recombination in *Saccharomyces cerevisiae* (66,67). β protein is required for phage λ recombination that is independent of RecA in *E. coli* (see (64)). If one considers these nonfilament-forming proteins, the stabilization of nascent homologous joints by RecA/Rad51 filaments can also be accomplished by other factors.

Nevertheless, filament forming and ATP-dependent RecA/Rad51-family recombinases are conserved in all living creatures, suggesting that filament formation and ATP hydrolysis play essential roles in homologous recombination and/or cell proliferation. Thus, the possibility is raised that filament formation plays an unidentified but essential role. A single-molecule imaging study suggests that RecA nucleoprotein filament with multiple dsDNA-binding sites accelerates sequence homology search within three-dimensional domains of DNA (68). As another possible role, ATP-hydrolysis-dependent and filament-dependent activities may be involved in the selection of recombination partners in homologous recombination repair. As shown by PCR, RecA excludes primers paired to template DNA with mismatched bases through an ATP-hydrolysis-dependent activity (69). This activity could contribute to fidelity in homologous recombination repair. Further studies are required to solve these issues.

## Supplementary Material

Supplementary Data
