# Supplementary material for: Nonfilament-forming RecA dimer catalyzes homologous joint formation
Source: Nucleic Acids Res. 2018 Oct 4;46(20):10855–69. doi: 10.1093/nar/gky877 (PMC6237804; doi:10.1093/nar/gky877)
Supplement: Supplementary Data [file gky877_supplemental_files.pdf]

SUPPLEMENTARY DATA

Nonfilament-forming RecA dimer catalyzes homologous joint formation

Takeshi Shinohara[1,2,3 †], Naoto Arai [4 †], Yukari Iikura [1,2], Motochika Kasagi [1,3], Tokiha Masuda-Ozawa [1,3], Yuuki Yamaguchi [1,3], Kayo Suzuki-Nagata [5], Takehiko Shibata [1,2,3,6], Tsutomu Mikawa [1,3,5,7]

[1] Cellular & Molecular Biology Laboratory, RIKEN, 2-1 Hirosawa, Wako-shi, Saitama 351-0198, Japan
[2] RIKEN Center for Sustainable Resource Science, 2-1 Hirosawa, Wako-shi, Saitama 351-0198, Japan
[3] Department of Supramolecular Biology, Graduate School of Nanobiosciences, Yokohama City University, 1-7-29 Suehiro-cho, Tsurumi-ku, Yokohama, Kanagawa 230-0045, Japan
[4] Department of Applied Biological Science, Nihon University College of Bioresource Sciences, 1866 Kameino, Fujisawa-shi, Kanagawa 252-0880, Japan
[5] RIKEN Quantitative Biology Center, 1-7-22 Suehiro-cho, Tsurumi-ku, Yokohama, Kanagawa 230-0045, Japan
[6] Department of Chemistry, Graduate School of Science, Tokyo Metropolitan University, Minami-Osawa 1-1, Hachioji-shi, Tokyo 192-0397, Japan
[7] RIKEN Center for Biosystems Dynamics Research, 1-7-22 Suehiro-cho, Tsurumi-ku, Yokohama, Kanagawa 230-0045, Japan

[†] The authors wish it to be known that, in their opinion, the first 2 authors should be regarded as joint First Authors.

Supplementary Materials and Methods

Expression of RecA variants

To express the RecA variants, *E. coli* BL21 ΔrecA (DE3) cells were transformed with the pET3a-npRecA monomer, the pET3a-npRecA dimer, and the pET3a-RecA-$wt_{\Delta C}$ vectors. For pET3a-RecA-$3m_{\Delta C}$, *E. coli* BL21 ΔrecA (DE3) pLysE cells were used. The transformed cells were cultured at 37°C in LB medium (1 L) containing ampicillin (50 µg/mL) to an OD_{600} of 0.4, as measured with a spectrophotometer. Isopropyl β-D-thiogalactopyranoside (IPTG) was then added to 0.5 mM (final concentration, unless otherwise stated), followed by incubation at 37°C for 5 h. Cells were harvested, frozen by dipping into liquid N_2 and stored at -25°C.

Additional information about the RecA-wt preparation, and the purification of RecA variants other than npRecA dimer

RecA-wt (*wild-type* RecA)

The protein was dialyzed against buffer containing Tris-HCl (20 mM; pH 7.5), EDTA (1 mM), DTT (5 mM) and 60% (w/v) glycerol and stored at -25°C. Care was taken to avoid oxidation of the RecA preparations (see ref. 21). The concentration of the RecA-wt was determined using $E^{1\,M}_{277nm}$ =24,000 (70).

Purification of the RecA variants was monitored by poly-acrylamide gel electrophoresis under denaturing conditions, including SDS and heating, as described previously (21). As a reference for molecular mass, we used SDS-PAGE Molecular Weight Standards, Low Range, manufactured by BIO-RAD (cat. No. 161-0305).

NpRecA monomer

The npRecA monomer was purified using a procedure similar to that used for the npRecA dimer, up to the P11 column-chromatography step. After elution of the npRecA monomer from the P11 column, the peak fractions were collected. KCl was then added to the suspension to a final concentration of 300 mM, and the protein was concentrated using an Amicon Ultra-15 10K filter (Merck Millipore). The concentrated protein was applied to a Superdex S-100 column (GE Healthcare) and eluted with TEMG5-K300 buffer (TEMG5 supplemented with KCl [300 mM]). The peak fractions were collected, concentrated, dialyzed against buffer containing Tris-HCl (20 mM; pH 7.5), EDTA (1 mM), DTT (5 mM), 60% (w/v) glycerol and KCl (300 mM) and stored at -25°C. The concentration of the npRecA monomer was determined using $E^{1\,M}_{280nm}$ =21,840.

RecA-$wt_{\Delta C}$ protein

The RecA-$wt_{\Delta C}$ protein was purified using a procedure similar to that used for the npRecA dimer, up to the P11 column-chromatography step. The RecA-$wt_{\Delta C}$ eluted from the P11 column was concentrated as described above, dialyzed against buffer containing Tris-HCl (20 mM; pH 7.5), EDTA (1 mM), DTT (5 mM) and 60% (w/v) glycerol and stored at -25°C. The concentration of the npRecA monomer was determined using $E^{1\,M}_{280nm}$ =21,840.

RecA-$3m_{\Delta C}$ protein

The RecA-$3m_{\Delta C}$ protein was purified using a procedure similar to that used for the npRecA dimer, but

the Polymin P treatment was omitted. The concentration of ammonium sulfate used on the TOYOPEARL Butyl-650M column was also changed, from 15% saturation to 20% saturation. Nuclease-free RecA-3m$_{\Delta C}$ was stored in Tris-HCl (20 mM; pH 7.5) containing EDTA (1 mM), DTT (0.1 mM) and 50% (w/v) glycerol at -25°C. The concentration of the npRecA monomer was determined using $E^{1\,M}{}_{280nm}$ =21,840.

Circular dichroism spectra

The CD spectra of the proteins (10 μM) were measured at 25°C in Tris-HCl (50 mM; pH 7.5) buffer containing EDTA (0.1 mM) and KCl (100 mM) with a Jasco spectropolarimeter, model J-820, as described previously (35).

Gel-filtration profile

The npRecA monomer and npRecA dimer were dialyzed against Tris-HCl buffer (25 mM; pH 7.5) containing EDTA (1 mM), DTT (0.1 mM) and KCl (150 mM), and adjusted to 10 μM using the same buffer. The RecA variants (100 μL) were then applied to a Superdex200 10/300 GL column (1 x 30 cm; GE Healthcare).

Electrophoretic mobility-shift assay for ssDNA-binding activity of RecA variants

6-fluorescein amidite (FAM) -labeled 90-mer OL2 ssDNA (4.5 μM) or unlabeled pUC119 dsDNA linearized by *Hin*dIII (9.0 μM) was incubated with a RecA variant and ATP or ATPγS (0 or 1.3 mM) at 37°C for 5 min or 10 min in the standard reaction mixture. When ATP was added, the reaction buffer was supplemented with an ATP-regeneration system, consisting of phosphocreatine (5 mM) and creatine phosphokinase (8 U/μL; Sigma-Aldrich Co. LLC.). Protein-bound DNA and free DNA were separated without fixation by electrophoresis through an agarose gel (1.2% for ssDNA, 1.0 % for dsDNA). The fluorescence from FAM of ssDNA and ethidium bromide-stained dsDNA was detected with a Typhoon FLA 9500 biomolecular imager (GE Healthcare).

DFM (Scanning-probe microscopy in dynamic force mode) observation of the npRecA dimer-ssDNA complex

RecA (final 0.2 μM) and npRecA dimer (final 0.2 μM) were added to a reaction buffer (final 20 μL) containing ΦX174 circular ssDNA (0.8 μM), ATPγS (0.5 mM), $MgCl_2$ (1.1mM), Tris-HCl (30 mM; pH 7.5) and DTT (1 mM) and incubated for 30 min at 37˚C. The reaction mixture (20 μL) was dropped onto a mica surface (1 cm x 1 cm) and treated as described for DFM of the npRecA dimer-dsDNA complex in Materials and Methods in the main text.

Standard assay for ATP/dATP hydrolysis by RecA

For ssDNA-dependent hydrolysis, [α-^{32}P] ATP (ribo-ATP) or [α-^{33}P] deoxyribo-ATP (dATP; 1.3 mM) was incubated at 37°C in the standard reaction buffer (20 μL), containing M13mp19 ssDNA (10 μM), with the indicated concentrations of RecA for the indicated times. For DNA-independent hydrolysis, [α-^{32}P] ATP or [α-^{33}P] dATP (1.3 mM) was incubated at 37°C in the standard reaction buffer (20 μL), containing sodium acetate (1.5 M), with the indicated concentrations of RecA, for the indicated times. The reactions were terminated by adding an equal amount of EDTA (25 mM) and left on ice. The samples were separated by thin layer chromatography, as previously described (21,33). The amount of α-^{32}P-labeled ATP and ADP, or α-^{33}P-labeled dATP and dADP, was determined with a BAS-2500

image analyzer (GE Healthcare), and the amount (nmol) of ATP or dATP hydrolyzed in a reaction mixture (20 μL) was calculated.

For RecA-3m$_{\Delta C}$ and the control RecA, unlabeled ATP was incubated in the presence of sodium acetate (1.5 M) as describe above, and the ATP-hydrolysis reaction was terminated by adding EDTA (final 5 mM) and SDS (final 5%) on ice water. Each sample was then diluted (10 fold) to 200 μL by adding H_2O at room temperature, followed by dilution by H_2O (60 fold). The residual amount of ATP in each diluted sample (50 μL) was quantified by measuring luminescence associated with luciferase-catalyzed mono-oxygenation of luciferin using CellTiter-Glo® reagent (50 μL; Promega) and a luminometer (Fluoroskan Ascent, Thermo Scientific).

Details of D-loop assay for homologous joint formation by RecA

[^{33}P]oligo-ssDNA (0.05 μM) and the indicated amounts of RecA variant were incubated for 5–15 min at 37°C in a standard buffer containing dATP or ATPγS (1.3 mM) instead of ATP, after which joint formation was initiated by the addition of homologous negatively supercoiled dsDNA (18 μM). Temperature shift during this procedure should be avoided, since at low temperatures (on ice), the activated RecA filament is inactivated (71).

The reaction buffer was supplemented with an ATP regeneration system consisting of phosphocreatine (5 mM) and creatine phosphokinase (8 U/μL; Sigma-Aldrich Co. LLC.). After incubation at 37°C for the indicated times, a portion (6 μL) of the reaction solution was removed, mixed with a stop solution (EDTA [66.7 mM] and 5% [w/v] SDS solution; 2.6 μL) and Proteinase K (2.4 mg/mL), then incubated on ice for 15 min. The samples were analyzed by electrophoresis using a 1.0% (w/v) agarose gel in 0.5x TBE buffer (Tris [25 mM; pH 8.0], boric acid [24 mM] and EDTA [1 mM]) and the ^{33}P-signals were analyzed with a BAS-2500 image analyzer.

D-loop assay for branch migration by RecA

In a D-loop assay, RecA-mediated branch migration, in the presence of ATP/dATP, is observed as ATP/dATP-hydrolysis-dependent dissociation of the homologous joints that form during the first phase of the reaction (see Fig. 5B, RecA-wt with dATP, and Fig. S2A and B). This dissociation is observed when homologous joints are formed by RecA with ssDNA of a limited length. The homologous joints (D-loops) formed on closed circular DNA move in a 5' to 3' direction of the displace strand by dsDNA-unwinding activity of RecA in an ATP-hydrolysis-dependent manner. When the trailing end of a D-loop reaches the 3' end of the paired ssDNA, the ssDNA is released from the D-loop (71-73; see ref. 21). *In vivo*, the migration of the D-loops associates with repair DNA synthesis initiated at the 3' end of the paired ssDNA (bubble migration; (see ref. 5 for references).

Strand exchange assay

Circular ssDNA (10 μM; M13mp18, TAKARA) and the RecA variant (5 μM) were incubated at 37°C for 15 min in the presence of $MgCl_2$ (1 mM), dATP (1.3 mM) and the ATP regenerating system that we confirmed to regenerate dATP. The lower concentration of $MgCl_2$ removed the requirement for SSB in the presynaptic phase (39). $MgCl_2$ was added to the reaction mixture to adjust the concentration to 13 mM, and the solution was incubated at 37°C for 5 min. To start the strand-exchange reaction, *Hin*cII-treated linear dsDNA (20 μM; M13mp18, TAKARA) and *E. coli* SSB (1 μM) were added to the reaction mixture, since SSB is known to prevent the formation of multimeric DNA complexes that suppress product yield (74). It is important to avoid temperature shifts during the reaction process. After incubation for the indicated times at 37°C, the reactions were terminated and the products were

fractionated by gel-electrophoresis, as described above.

The gel was stained with ethidium bromide (1 μg/mL) and photographed with UV illumination at 312 nm. Smart Ladder (from top to bottom: 10, 8, 6, 5, 4, 3, 2.5, 2, 1.5, 1.0, 0.80 and 0.60 kbp; Nippon Gene) markers were used as DNA size markers. Black and white of the electrophoresis images were inverted using ImageJ software (https://imagej.nih.gov/ij/).

Fluorometric εssDNA unstacking assay

Etheno-modified ssDNA (εssDNA) was calf-thymus-DNA fragmented to a length of about 300 mer, as described previously (35,75). We measured the fluorescence emitted by 1,N^6-ethenoadenine in εssDNA upon excitation at 305 nm, as previously described (75). We incubated the RecA variants (1 μM) with or without ATPγS (0.2 mM), in Tris-HCl buffer (25 mM; pH 7.5) containing $MgCl_2$ (10 mM) and DTT (1 mM), at 25°C for 5 min, then added various amounts of εssDNA. The fluorescence was measured at 25°C with a fluorescence spectrometer equipped with a temperature-regulated cuvette holder.

References for Supplementary Data

70. Tsang, S.S., Muniyappa, K., Azhderian, E., Gonda, D.K., Radding, C.M., Flory, J. and Chase, J.W. (1985) Intermediates in homologous pairing promoted by recA protein: isolation and characterization of active presynaptic complexes. *J. Mol. Biol.*, **185**, 295-309.
71. Shibata, T., Ohtani, T., Iwabuchi, M. and Ando, T. (1982) D-loop cycle: a circular reaction sequence which comprises formation and dissociation of D-loops and inactivation and reactivation of superhelical closed circular DNA promoted by recA protein of *Escherichia coli*. *J. Biol. Chem.*, **257**, 13981-13986.
72. Ohtani, T., Shibata, T., Iwabuchi, M., Watabe, H., Iino, T. and Ando, T. (1982) ATP-dependent unwinding of double helix in closed circular DNA by recA protein of *E. coli*. *Nature*, **299**, 86-89.
73. Wu, A.M., Kahn, R., DasGupta, C. and Radding, C.M. (1982) Formation of nascent heteroduplex structures by RecA protein and DNA. *Cell*, **30**, 37-44.
74. Chow, S.A., Rao, B.J. and Radding, C.M. (1988) Reversibility of strand invasion promoted by recA protein and its inhibition by *Escherichia coli* single-stranded DNA-binding protein or phage T4 gene 32 protein. *J. Biol. Chem.*, **263**, 200-209.
75. Menetski, J.P. and Kowalczykowski, S.C. (1985) Interaction of recA protein with single-stranded DNA. Quantitative aspects of binding affinity modulation by nucleotide cofactors. *J. Mol. Biol.*, **181**, 281-295.
76. Zlotnick, A., Mitchell, R.S., Steed, R.K. and Brenner, S.L. (1993) Analysis of two distinct single-stranded DNA binding sites on the recA nucleoprotein filament. *J. Biol. Chem.*, **268**, 22525-22530.

Supplementary Figures

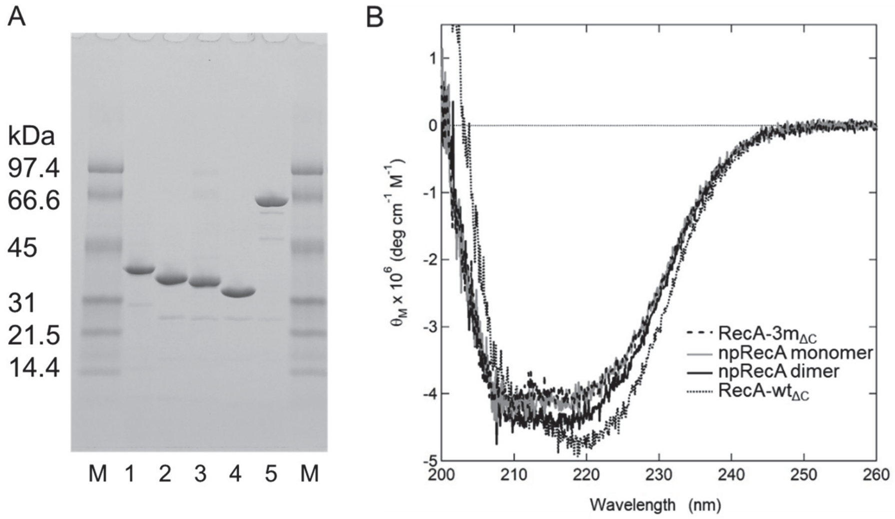

Figure S1. Purity, peptide size and folding of RecA variants

A. SDS polyacrylamide gel-electrophoretic profiles of purified RecA variants.

Purified RecA variants (0.7 µg) were analyzed under denaturing conditions, including SDS. Lane 1 shows RecA-wt (37.9 kDa); lane 2 shows RecA-wt$_{\Delta C}$ (36.2 kDa); lane 3 shows RecA-3m$_{\Delta C}$ (36.2 kDa); lane 4 shows the npRecA monomer (32.9 kDa); and lane 5 shows the npRecA dimer (70.4 kDa). The M lanes show the size markers (97.4, 66.6, 45, 31, 21.5 and 14.4 kDa).

B. Circular dichroism spectra of RecA variants.

RecA variants (10 µM) were analyzed by circular dichroism spectrometry. At 220 nm in the spectrum of the npRecA monomer, intensity was slightly decreased, compared with that of the RecA-wt$_{\Delta C}$ control. This reflects a decrease in the α helical content and must be due to the lack of an N-terminal domain. As expected, the npRecA dimer, which retains only one of two N-terminal domains, had an intensity at 220 nm between that of the RecA-wt$_{\Delta C}$ control and the npRecA monomer. The CD spectrum of the RecA-3m$_{\Delta C}$ variant coincided with that of the npRecA monomer, suggesting that the N-terminal domain is unstructured. Thus, although RecA-3m$_{\Delta C}$ forms oligomers (Fig. 2B), the inter-protomer interface is abnormal (see ref. 35).

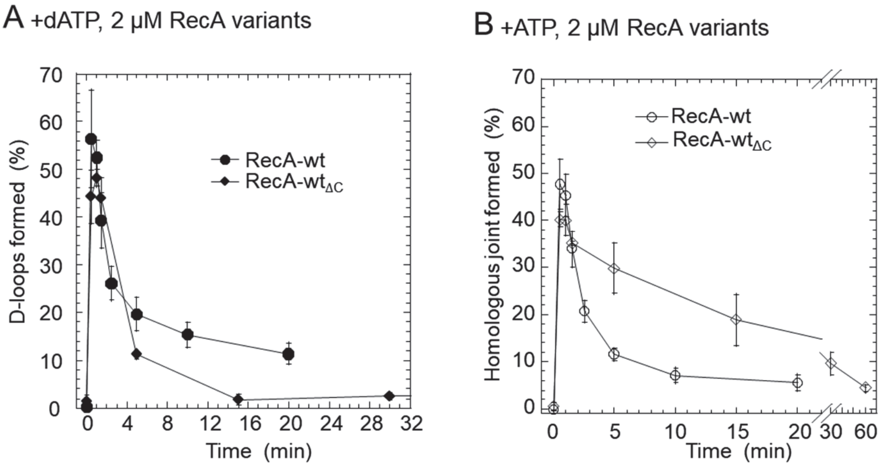

Figure S2. Comparison of RecA-wt and RecA-wt$_{\Delta C}$.

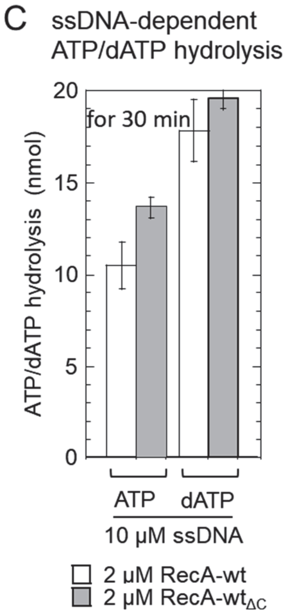

A and B. Homologous joint formation and branch migration as observed by D-loop dissociation.

90-mer [^{33}P] oligo (OL2; 0.05 µM**)** and RecA **(**2 µM**)** were incubated in the presence of dATP (A) or ATP (B) in a standard reaction buffer at 37°C for 5-15 min, after which homologous negatively supercoiled dsDNA **(**18 µM**;** pBluescript SK(-)) was added to initiate the reaction. The mixtures were incubated at 37°C for the indicated times. The values obtained for RecA-wt in the presence of dATP were the same as those shown in Fig. 5B in the main text. Black symbols represent the presence of dATP; white symbols represent the presence of ATP. Circles indicate RecA-wt and OL2 ssDNA and diamonds indicate RecA-wt$_{\Delta C}$ and OL2 ssDNA. Results obtained by strand-exchange assay (Fig. S5) also show that RecA-wt$_{\Delta C}$ is active in homologous joint formation and branch migration.

C. ssDNA-dependent ATP/dATP hydrolysis

The amount of ATP/dATP hydrolyzed during incubation (30 min) in the presence of circular ssDNA (10 µM) and RecA-wt or RecA-wt$_{\Delta C}$ (2.0 µM) in a standard reaction buffer was plotted. White bars indicate RecA-wt; gray bars indicate RecA-wt$_{\Delta C}$. As in Fig. 4F, the observed amounts of ADP and dADP in the controls (without DNA) were subtracted from the values obtained in the presence of ssDNA for each experiment. The control values were 0.8 and 0.2 nmol for ADP and dADP, respectively, in the presence of RecA-wt, and 0.8 and 0.3 nmol for ADP and dADP, respectively, in the presence of RecA-wt$_{\Delta C}$.

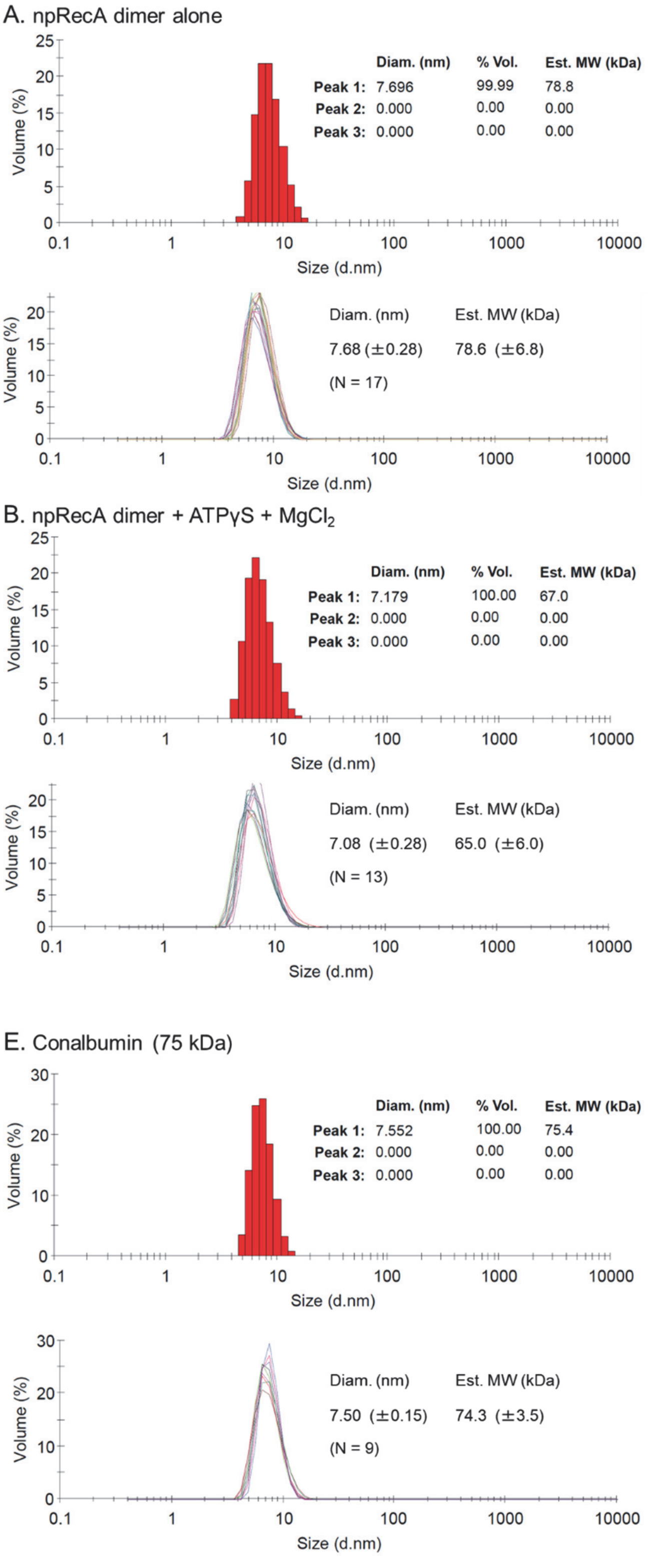

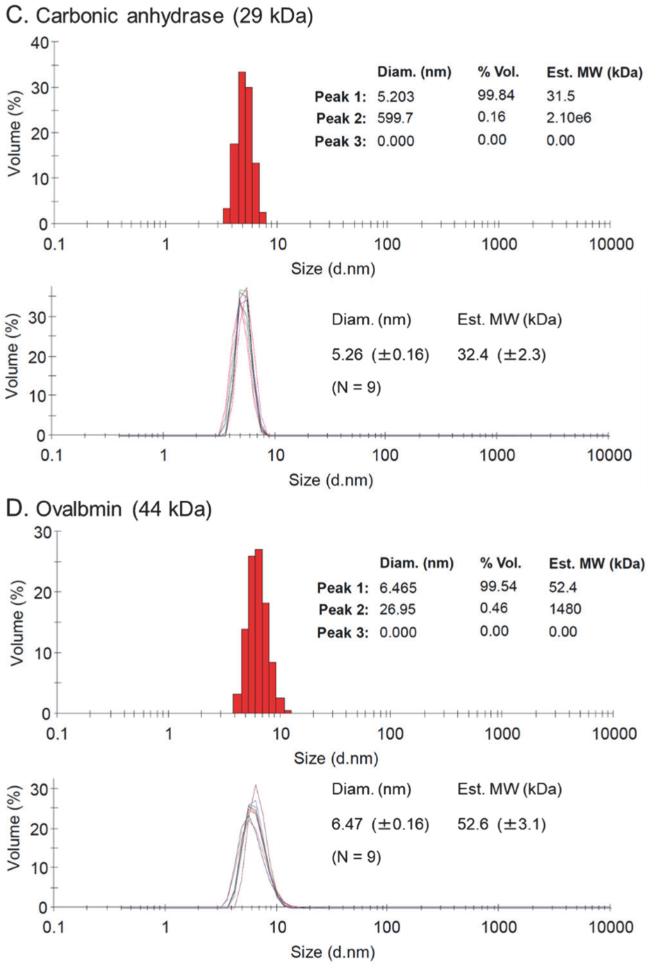

Figure S3. Dynamic light-scattering data of the RecA dimer and controls.

We analyzed the npRecA dimer in the absence (A) and presence (B) of ATPγS and Mg^{2+} along with molecular mass control proteins, carbonic anhydrase (29 kDa; C), ovalbumin (44 kDa; D), and conalbumin (75 kDa; E). See Table 1 for conditions. The upper panel and bottom panel of each set represent an example of an analytical result and overlaid results of all analysis, respectively, for the diameter of the molecule in each series of measurements.

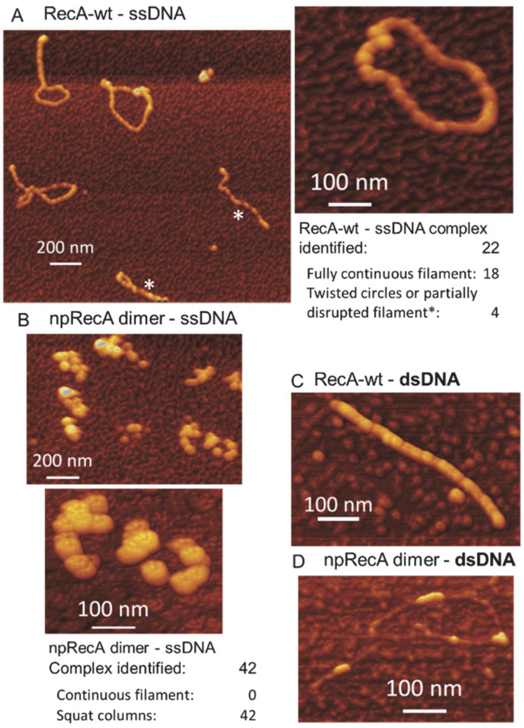

Figure S4. DFM analysis of npRecA dimers after incubation with circular ssDNA.

RecA-wt (A) and npRecA dimers (B) at 0.2 μM (RecA subunit) were allowed to form complexes with phage ΦX174 circular ssDNA (0.8 μM) in the presence of ATPγS (1 mM) and $MgCl_2$ (1.1 mM). The complexes were fixed with glutaraldehyde (0.2%) on a mica surface and visualized by DFM (a scanning-probe microscope in dynamic force mode).

For the comparison, the RecA-wt-dsDNA complex (C) and the npRecA dimer-dsDNA complex (D) prepared in the experiments shown in Fig. 3 are presented.

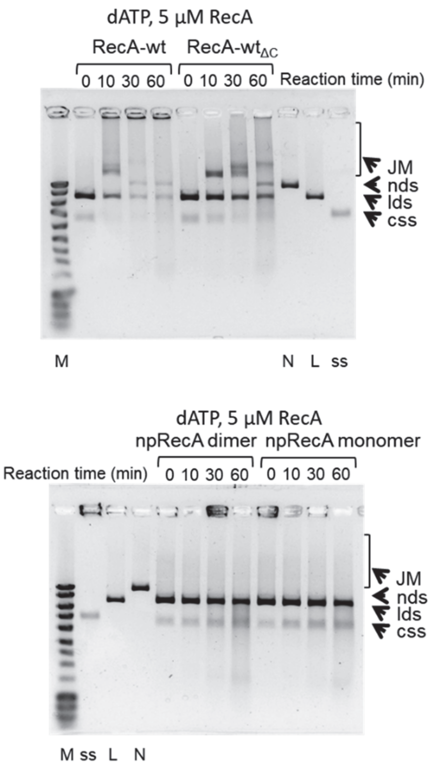

Figure S5. Strand-exchange assay for RecA variants in the presence of dATP.

Circular ssDNA (10 μM) was incubated with the indicated RecA variants (5 μM) for 15 minutes, in the presence of $MgCl_2$ (1 mM) and dATP. Subsequently, $MgCl_2$ was increased to 13 mM. After 5 min, homologous joint formation was initiated by the addition of homologous linear dsDNA-substrate (20 μM). *E. coli* SSB was added simultaneously, followed by incubation for the indicated times. css = circular ssDNA; lds = linear dsDNA; nds = nicked circular dsDNA; JM = joint molecules. Lane N shows nds (M13 mp18); lane L shows lds substrate; lane ss shows css substrate; lane M shows the DNA markers (Smart Ladder, Nippon Gene; 10,000, 8,000, 6,000, 5,000, 4,000, 3,000, 2,500, 2,000, 1,500, 1,000, 800, 600, 400, 200 bp).

RecA-wt formed homologous joints, detected initially as joint molecules and then as nicked circular dsDNA, the final product of branch migration. We replaced SSB by incubation with $MgCl_2$ (1 mM) during the binding of the npRecA dimer to the ssDNA (39) to eliminate possible competition by SSB in ssDNA binding. But after $MgCl_2$ (to 13 mM) and dsDNA had been added to initiate homologous joint formation, we reintroduced SSB to prevent the formation of multimeric DNA complexes (74).

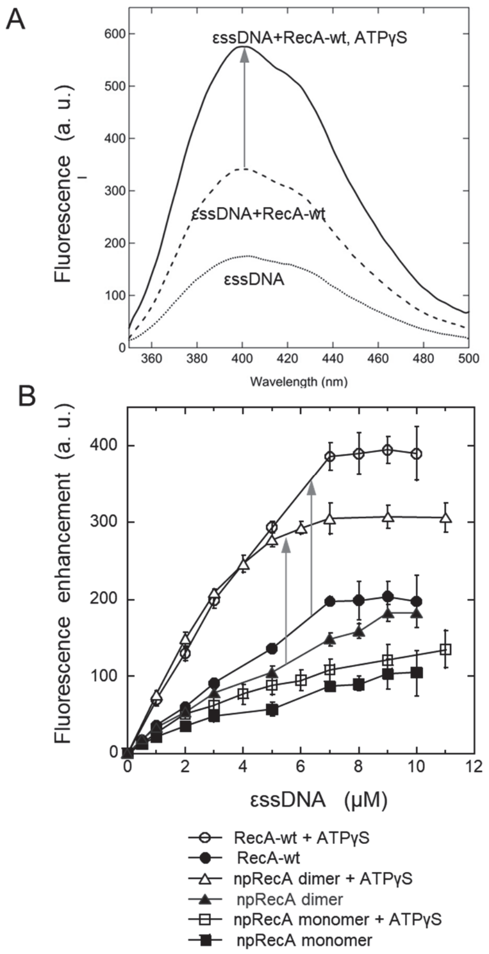

Figure S6. Base unstacking of ssDNA by RecA variants, as shown by use of εssDNA.

A. Fluorescence-emission spectra of εssDNA in the presence or absence of RecA-wt and ATPγS.

Fluorescence-emission spectra were measured in the presence of εssDNA (7 μM), with or without RecA-wt (1 μM) and ATPγS (0.2 mM), at 25°C. Fluorescence is expressed in arbitrary units (a. u.).

B. Relief of fluorescence quenching of εssDNA by RecA variants in the presence or absence of ATPγS.

Fluorescence at 400 nm was measured at 25°C in the presence of RecA variant (1 μM) and the indicated concentration of εssDNA, with or without ATPγS. Fluorescence enhancement was defined as the increased fluorescence above that of protein-free εssDNA. Circles represent RecA-wt, triangles represent npRecA dimers and squares represent npRecA monomers. White symbols represent the presence of ATPγS and black symbols represent the absence of ATPγS.

Fluorescence enhancement of εssDNA was examined in the presence of a fixed amount of RecA (1 μM) with varying amounts of DNA in the presence or absence of ATPγS. Fluorescence enhancement by RecA-wt was proportional to the amount of ssDNA added, while the addition of ATPγS increased fluorescence enhancement by about 2 fold (A and B), as previously reported (76). In this assay, although an increase in fluorescence enhancement by the addition of ATPγS was not observed, the addition of the npRecA monomer did result in an increase in the fluorescence of εssDNA, indicating the presence of a nonspecific interaction.
